# Geometry and the Organizational Principle of Spine Synapses along a Dendrite

**DOI:** 10.1523/ENEURO.0248-20.2020

**Published:** 2020-12-17

**Authors:** Laxmi Kumar Parajuli, Hidetoshi Urakubo, Ai Takahashi-Nakazato, Roberto Ogelman, Hirohide Iwasaki, Masato Koike, Hyung-Bae Kwon, Shin Ishii, Won Chan Oh, Yugo Fukazawa, Shigeo Okabe

**Affiliations:** 1 Department of Cellular Neurobiology, Graduate School of Medicine, The University of Tokyo, Tokyo 113-0033, Japan; 2 Department of Cell Biology and Neuroscience, Juntendo University Graduate School of Medicine, Tokyo 113-8421, Japan; 3 Department of Systems Science, Graduate School of Informatics, Kyoto University, Kyoto 606-8501, Japan; 4 Department of Pharmacology, University of Colorado School of Medicine, Aurora, CO 80045; 5 Department of Anatomy, Graduate School of Medicine, Gunma University, Gunma 371-8511, Japan; 6 Max Planck Florida Institute for Neuroscience, Jupiter, FL 33458; 7 Department of Neuroscience, Johns Hopkins School of Medicine, Baltimore, MD 21205; 8 Division of Brain Structure and Function, Research Center for Child Mental Development, Life Science Advancement Program, University of Fukui, Fukui 910-1193, Japan

**Keywords:** dendritic spine, electron microscopy, FIB/SEM, glutamate uncaging, postsynaptic density, simulation

## Abstract

Precise information on synapse organization in a dendrite is crucial to understanding the mechanisms underlying voltage integration and the variability in the strength of synaptic inputs across dendrites of different complex morphologies. Here, we used focused ion beam/scanning electron microscope (FIB/SEM) to image the dendritic spines of mice in the hippocampal CA1 region, CA3 region, somatosensory cortex, striatum, and cerebellum (CB). Our results show that the spine geometry and dimensions differ across neuronal cell types. Despite this difference, dendritic spines were organized in an orchestrated manner such that the postsynaptic density (PSD) area per unit length of dendrite scaled positively with the dendritic diameter in CA1 proximal stratum radiatum (PSR), cortex, and CB. The ratio of the PSD area to neck length was kept relatively uniform across dendrites of different diameters in CA1 PSR. Computer simulation suggests that a similar level of synaptic strength across different dendrites in CA1 PSR enables the effective transfer of synaptic inputs from the dendrites toward soma. Excitatory postsynaptic potentials (EPSPs), evoked at single spines by glutamate uncaging and recorded at the soma, show that the neck length is more influential than head width in regulating the EPSP magnitude at the soma. Our study describes thorough morphologic features and the organizational principles of dendritic spines in different brain regions.

## Significance Statement

Little is known about the characteristic anatomic features underlying the organization of spine synapses in a dendrite. This study used volume electron microscopy to make an extensive characterization of dendritic spine synapses in multiple regions of the mouse brain to uncover the principles underlying their placement along a dendritic shaft. By using a combination of approaches such as two-photon imaging, glutamate uncaging, electrophysiology, and computer simulation, we reveal the functional importance of regulated spine placement along a dendritic trunk. Our research presents a crucial step in understanding the synaptic computational principle in dendrites by highlighting the generalizable features of dendritic spine organization in a neuron.

## Introduction

Neurons communicate with each other via synapses. In multiple brain regions, synaptic communications occur through tiny dendritic protrusions called dendritic spines (Parajuli et al., 2017). Evidence suggests that the placement of synapses in a dendrite occurs in a regulated manner (Jones and Powell, 1969; Magee and Cook, 2000; Konur et al., 2003; Katz et al., 2009; Menon et al., 2013; Walker et al., 2017). The plastic nature of dendritic spines (Matsuzaki et al., 2004) and their ability to cross talk with each other (Royer and Paré, 2003; Oh et al., 2015) hint at the possibility that dendritic spines are organized to maintain a set of optimal rules.

Neurons from different brain regions are distinct in terms of the gross anatomic features. In addition, a single neuron shows considerable variability in the size and shape of dendritic arbors (Harris and Spacek, 2016; Parajuli et al., 2020a), thereby creating variation in the electrical properties among different dendritic compartments. Thus, defining the spatial organizational principle of synapses in a dendrite is key to understanding how the mechanism of voltage integration differs along the somatodendritic arbor in diverse neurites. Furthermore, there has recently been an enormous surge of interest in mapping each of the neuronal connections in the brain (Bock et al., 2011; Kasthuri et al., 2015; Markram et al., 2015). However, even with the latest automated large-scale electron microscopic (EM) techniques, it is impractical to achieve comprehensive sorting of individual neuron-to-neuron connections. Thus, in recent years investigators feel the urge to minimize the effort and cost involved in connectomics studies by studying a handful of brain regions and extracting some common organizational principles for neuronal connections in those regions. Decoding principles of synapse placement in a handful of brain regions might eventually aid in formulating a set of algorithms that apply to most brain nuclei.

Studies in the past have used conventional transmission EM (TEM) to make both qualitative and quantitative descriptions of dendritic spines (Wilson et al., 1983; Harris and Stevens,1988, 1989; Harris et al., 1992; Schikorski and Stevens, 1997). Several past studies are limited to single section observations. Even with the approach of serial sectioning, imaging volume is often small because the manual collection and image acquisition of hundreds of thin sections is technically demanding. Most of the previous studies did not attempt to study spines in relation to their parent dendrites. A survey of the spine position along the dendritic arbor can reveal how synaptic conductance scales up to counterbalance the effect of passive membrane properties in dendrites (Rall, 1962; Katz et al., 2009). Furthermore, no studies have yet been undertaken to compare and contrast the synapse structure between different brain regions and decipher which principles are universal and which aspects are intrinsic to a given brain region. It is not directly possible to compare the findings from different studies as there are inconsistencies in the results because of differences in the genetic background of animal models, sample preparation, and analysis methods.

Since synaptic junctions are too small to be resolved by light microscopy (Okabe, 2020), here we used focused ion beam/scanning EM (FIB/SEM) to perform automatic imaging of neuropils from multiple brain regions that exhibit numerous dendritic spines. By large volume reconstruction at the synaptic level resolution, we construct a quantitative description of the morphologic features of dendritic shafts, dendritic spines, and their presynaptic connectivity pattern, and reveal the principle defining dendritic spine organization in a dendrite. Using two-photon glutamate uncaging and electrophysiological recording, we show that the spine neck length is more influential than head width in regulating the magnitude of EPSPs at the soma. In summary, we report the organizational principle of dendritic spine synapses in their parent dendrites across multiple brain regions.

## Materials and Methods

### EM

#### Animals

A total of four C57BL/6 male mice at 12 weeks of age were used for this study. All the animals were raised in a standard light-dark cycle and had free access to food and water. The animal handling protocol was approved by the animal care and use committee of the authors’ institutions. Mouse housing and euthanization strictly adhered to the guidelines provided by the government and the university. Efforts were made to reduce the pain and suffering of the animals used in the experiment.

#### Tissue preparation for FIB/SEM analysis

Sample preparation for FIB/SEM was performed as described previously (Takahashi-Nakazato et al., 2019). Mice were anesthetized by intraperitoneal injection of somnopentyl (10 ml/kg body weight) and transcardially perfused with 20 ml of Ringer’s solution, followed by 70 ml of 2% paraformaldehyde and 2.5% glutaraldehyde made up in 0.1 m cacodylate buffer (pH 7.4). The brains were quickly removed and postfixed for 1 h at room temperature in 4% paraformaldehyde solution. After several washes in cacodylate buffer, 100-μm-thick sections were cut in ice-cold 0.1 m phosphate buffer (PB; pH 7.4) using a vibratome (Leica VT 1000S). Sections were washed in cacodylate buffer containing 2 mm calcium chloride. Sections were then incubated in a freshly prepared solution containing 3% potassium ferrocyanide (made up in 0.2 m cacodylate buffer and supplemented with 4 mm calcium chloride) combined with an equal volume of 4% aqueous osmium tetroxide. Sections were then washed in Milli-Q water (Millipore) and incubated at room temperature for 20 min in the thiocarbohydrazide solution. The solution was prepared by incubating 0.1 g of thiocarbohydrazide in 10 ml ddH_2_O in a 60°C oven for 1 h. The sections were then placed in a 2% aqueous osmium tetroxide solution for 30 min, washed briefly, and incubated overnight in 1% uranyl acetate. The sections were further stained with filtered Walton’s lead aspartate solution in a 60°C oven for 75 min and then dehydrated in ascending series of ethanol. Slices were placed in propylene oxide solution, gradually equilibrated with Durcupan resin, flat embedded, and placed in a 60°C oven for 48 h for resin curing and polymerization.

#### FIB/SEM imaging

Resin blocks containing the section of interest were mounted on metal stubs. The block was trimmed with a diamond knife to expose the resin tissue interface to the surface. We prevent specimen charging by painting the stub with graphene at block sides, and coating with a 5- to 7-nm-thick carbon layer over block faces using a BAF060 freeze-fracture replica machine (Leica). Aluminum stubs containing the specimen were placed in the FIB/SEM stage (FEI), and images were acquired at an acceleration voltage of 1.4 kV, dwell time: 5 μs and z-step: 40 nm. Images were taken at 17,500× magnification, covering a horizontal field width of 11.84 μm at a resolution of 3072 × 2048 pixels. Automated acquisition of 200–450 serial images was performed by the sequential repetition of sample milling and imaging using Auto Slice and View G3 software (FEI).

FIB/SEM images were acquired from the proximal stratum radiatum (PSR) and stratum lacunosum-moleculare (SLM) of hippocampal CA1, PSR of hippocampal CA3, layer 1 of the somatosensory cortex, striatum, and molecular layer of the cerebellum (CB).

### Three-dimensional (3D) reconstruction and quantitative analysis of neuropil from FIB/SEM images

Images were first aligned with the aid of Fiji software (Schindelin et al., 2012) and then loaded onto Reconstruct software (Fiala, 2005) for manual segmentation of the neuronal profiles of interest. Volume reconstruction and measurement of the profile dimensions were performed using Reconstruct. Only a complete spine whose head and neck were contained within the imaging volume was analyzed. The spine head volume was obtained by multiplying the total cross-sectional area of the plasma membrane contours with the section thickness. Postsynaptic density (PSD) area in a cross-sectioned synapse was calculated by multiplying section thickness with the summed length of PSD in consecutive sections. For synapses cut en face, the PSD area was obtained in the single section. Dendritic length, neck length, and neck diameter were measured in 3D reconstructed images in reference to the 1 μm^3^ scale cube. The dendritic diameter was obtained by computing the average length of the line that was drawn across the widest transect of the narrowest dimension of the ovoid profile. Spine density was obtained by dividing the number of spines in a dendrite by the length of the reconstructed dendrite. PSD area density (expressed as the PSD area per unit length of the dendrite) was obtained by dividing the summed PSD area of all the spines in a dendritic segment by the length of the dendrite. Occasionally, some spine necks extended beyond the field of view, and this prevented us from measuring the PSD area in those spines. Thorny excrescence spines in CA3 were only used as an example to show their vast difference in spine morphology from that of typical spines in other brain regions. We did not perform any quantification of the synaptic density and neck length of CA3 thorny excrescence spines. In the CB, quantitative analysis was exclusively performed from Purkinje cell spines that made synaptic contacts with the parallel fiber terminals. Climbing fiber–Purkinje cell synapses were rarely observed in our FIB/SEM images.

### Pre-embedding immunogold labeling

Pre-embedding immunogold labeling was conducted as described in Parajuli et al. (2020b). We perfused a mouse with 75 ml of fixative containing 4% PFA and 0.05% glutaraldehyde. Fifty-micrometer cryoprotected sections were freeze-thawed and incubated in a blocking solution containing 20% normal goat serum in 50 mm Tris-buffered saline. Sections were incubated for 48 h with 1 μg/ml of inositol 1,4,5-trisphosphate receptor type 1 (IP_3_R1) antibody (Frontier Institute) and then overnight in 1.4-nm gold-conjugated anti-rabbit secondary antibody (Nanoprobes). Immunoreactivity was visualized by placing sections in the HQ silver intensification solution (Nanoprobes). Sections were osmicated for 40 min, followed by incubation in 1% uranyl acetate for 35 min at room temperature. Sections were dehydrated in graded ethanol series of 50%, 70%, 80%, 90%, and 100% for 10 min each and incubated for another 10 min in propylene oxide to facilitate resin penetration. Sections were placed in freshly prepared resin overnight for resin infiltration and placed in a 60°C oven for 48 h for resin curing. Seventy-nanometer ultrathin sections were cut with a Leica Ultracut UCT ultramicrotome and collected on formvar-coated single-slot grids. After that, they were counterstained using Reynold’s lead citrate and imaged using a JEM-1010 TEM (JEOL). Single section images were randomly acquired from the cortex, striatum, and CB, and the frequency of immunogold positive spine profiles in each brain region was counted.

### Two-photon imaging, glutamate uncaging and electrophysiology

#### Preparation of hippocampal slices

Acute coronal hippocampal slices were prepared from C57BL/6 wild-type mice, postnatal day (P)30–P43. Mice were anesthetized with isoflurane and decapitated. The brain was removed from the skull and rapidly placed in an ice-cold cutting solution containing the following: 215 mm sucrose, 20 mm glucose, 26 mm NaHCO_3_, 4 mm MgCl_2_, 4 mm MgSO_4_, 1.6 mm NaH_2_PO_4_, 1 mm CaCl_2,_ and 2.5 mm KCl. Coronal slices (300 μm thick) were prepared using a VT1000S vibrating microtome (Leica). Slices were incubated at 32°C for 30 min in a holding chamber containing 50% cutting solution and 50% artificial CSF (ACSF; 127 mm NaCl, 25 mm NaHCO_3_, 1.25 mm NaH_2_PO_4_, 2.5 mm KCl, 25 mm D-glucose, 2 mm CaCl_2_, and 1 mm MgCl_2_). After 30 min, this solution was replaced with ACSF at room temperature. Slices were allowed to recover for >1 h in ACSF before imaging and recording. All solutions were equilibrated for at least 30 min with 95%O_2_/5%CO_2_. Organotypic hippocampal slice cultures were prepared from P3 mice, following the guidelines set by the institutional animal care and use committee at the authors’ institutions.

### Two-photon imaging, uncaging, and electrophysiology

CA1 pyramidal neurons were imaged using a two-photon microscope (Prairie Technologies, Inc) with a pulsed Ti:sapphire laser (MaiTai HP DeepSee, Spectra-Physics) tuned to 920 nm (3–4 mW at the sample) in recirculating ACSF aerated with 95%O_2_/5%CO_2_ containing the following: 0.001 mm TTX and 2.3–2.5 mm MNI-caged-glutamate. For each neuron, image stacks (512 × 512 pixels; 0.035 μm/pixel) with 1 μm z-steps were collected from several secondary or tertiary apical and/or basal dendrites (average of three dendrites per cell) located 40–80 μm from the soma. All images shown are maximum projections of 3D image stacks after applying a median filter (2 × 2) to the raw image data. Uncaging of MNI-glutamate was achieved as described (Oh et al., 2016). In brief, whole-cell recordings (electrode resistance 6–8 MΩ; series resistance 20–40 MΩ) were performed at 30°C on visually identified CA1 pyramidal neurons using a MultiClamp 700B amplifier (Molecular Devices). In order to record uncaging-evoked EPSCs (uEPSCs), CA1 pyramidal neurons of acute hippocampal slices at depths of 20–40 μm were patched in voltage-clamp configuration (V_hold_ = −65 mV for AMPA receptor-mediated uEPSCs) using cesium-based internal solution (135 mm Cs-methanesulfonate, 10 mm HEPES, 10 mm Na_2_-phosphocreatine, 4 mm MgCl_2_, 4 mm Na_2_-ATP, 0.4 mm Na-GTP, 3 mm Na L-ascorbate, 0.2 mm Alexa Fluor 488; ∼300 mOsm, ∼pH 7.25) in ACSF. Uncaging EPSPs (uEPSPs) were measured from 28 d *in vitro* (DIV) CA1 pyramidal neurons of organotypic slice cultures in current-clamp configuration (I = 0) using potassium-based internal solution (136 mm K-gluconate, 10 mm HEPES, 17.5 mm KCl, 9 mm NaCl, 1 mm MgCl_2_, 4 mm Na_2_-ATP, and 0.4 mm Na-GTP; ∼300 mOsm, ∼pH 7.26) at 30°C in ACSF containing 2 mm Ca^2+^ and 1 mm Mg^2+^. uEPSPs were recorded from individual spines with different neck lengths and head sizes. uEPSC or uEPSP amplitudes from several spines (average of four spines per dendrite) that were well isolated from each other on a single dendritic segment were quantified as the average (5–10 test pulses of 2-ms duration at 0.1 Hz) from a 2-ms window centered on the maximum current amplitude after uncaging pulse delivery. Laser pulses were delivered by parking the beam at a point ∼0.5 μm from the center of the spine head with a pulsed Ti:sapphire laser (MaiTai HP, Spectra-Physics) tuned to 720 nm (17–20 mW at the sample).

### Quantification of fluorescence intensities of dendritic spines

Integrated green fluorescence intensities were measured from background-subtracted green fluorescence (Alexa Fluor 488 in internal solution) using the integrated pixel intensity of a boxed region surrounding the spine head. The estimated spine size was calculated by normalizing the fluorescence intensities for each spine on a single dendritic segment to the mean fluorescence intensities measured from four regions of interest (ROIs) on the dendritic shaft. Spine length/spine width ratio was obtained by dividing the spine length (measured from the tip of the spine head to the base of the spine neck) by the spine head width at its widest transect (Woods et al., 2011). For spines that show no discernible necks, we set a minimum length of 0.2 μm, as previously described (Araya et al., 2006b, 2014).

### Computer simulation study

#### Two-layer model neuron

We developed a two-layer model neuron that consists of 20 branches (*N_branch_* = 20) with each branch having 250 synapses (*N_syn_* = 250). If each synapse *i* at the branch *j* follows an input sequence {*t_i,j,1_*, *t_i,j,2_*, …, *t_i,j,k_*}, the total synaptic input to the branch *I_j_*(*t*) can be described by:

$$I_{j}\left(t\right)=\overset{N_{syn}}{\underset{i}{\sum }}\underset{k}{\sum }W_{i,j}\delta \left(t-t_{i,\;j,\;k}\right),$$

where *W_i,j_* is the synaptic weight, and *δ*(*t*) is the Dirac’s δ function. Here, the input sequence {*t_i,j,1_*, *t_i,j,2_*, …, *t_i,j,k_*} obeys a Poisson process with the input frequency ranging from 0.5 to 10 Hz. The synaptic weight *W_i,j_* was sampled from the lognormal distribution:

$$P_{W}\left(X\right)=\frac{1}{\sqrt{2\pi }\sigma_{e}X}\exp\;\left(-\frac{{\left(\log\;X-\mu_{e}\right)}^{2}}{2\sigma_{e}{}^{2}}\right),$$

where *μ_e_*and *σ_e_* respectively represent the location and shape parameters of the probability distribution. Dendritic membrane potential *V_j_* depolarized by the summed synaptic input *I_j_*(*t*) results in the occurrence of dendritic spikes as:

$$\frac{dV_{j}}{dt}=-\frac{\left(V_{j}-V_{rest}\right)}{\tau_{dend}}\;+\;I_{j}\left(t\right),$$

if $V_{j}\;>\;\theta$, then $V_{j}\to V_{reset,dend}$, where *V_rest_* represents the resting membrane potential, *V_reset,dend_* represents the dendritic reset potential, *τ_dend_* represents the dendritic membrane time constant, and *θ* represents the spiking threshold. In our simulation, we set *V_rest_* and *V_reset,dend_* to −75 mV, *τ_dend_* to 10 ms, and *θ* to −40 mV. A relative refractory period of 20 ms was imposed on the dendritic spikes that occur in the model dendritic branches.

Dendritic spike mediated depolarization of somatic membrane potential *V_m_* and firing of a somatic spike is expressed as:

$$\frac{dV_{m}}{dt}=-\frac{\left(V_{m}-V_{rest}\right)}{\tau_{soma}}+\overset{N_{branch}}{\underset{i}{\sum }}\underset{j}{\sum }W\delta \left(t-t_{i,j}^{dend}\right),$$

if $V_{m}\;>\;\theta$, then $V_{m}\to V_{reset,soma}$, where {*t_i,1_^dend^*, *t_i,2_^dend^*, …, *t_i,j_^dend^*} is the dendritic spike sequence from the branch *i*, *W* is the weight (the level of synaptic depolarization by a dendritic spike), *V_reset,soma_* is the somatic reset potential, and *τ_soma_* is the somatic membrane time constant. We set *W* to 20 mV, *τ_soma_*to 20 ms, and *V_reset,soma_* to −80 mV. The model soma had a relative refractory period of 20 ms. *τ_soma_* and *τ_dend_* were determined based on a previous study (Routh et al., 2009). The output somatic spike sequences {*t_1_^soma^*,*t_2_^soma^*, …, *t_i_^soma^*} were subjected to further analysis.

### Mutual information

First, we targeted the input sequence to the strongest synapse, *imax*, at each branch, *j*, {*t_imax,j,1_*, *t_imax,j,2_*, …, *t_imax,j,k_*}. The input sequence was binned to 5-ms intervals and binarized [synaptic input (*SI*) =* *0 for the absence of synaptic input; *SI *=* *1, for the occurrence for input]. Information transfer by the occurrence of dendritic spikes {*t_i,1_^dend^*, *t_i,2_^dend^*, …, *t_i,j_^dend^*} within the same time bins [dendritic spike (*DS*) =* *0 for the absence of dendritic spike; *DS *=* *1 for the occurrence of dendritic spike] were quantified using mutual information (MI):

$$\text{MI}\left(DS,SI\right)=\underset{DS\in \{0,1\}}{\sum }\underset{SI\in \{0,1\}}{\sum }p(DS,SI)\log\;\frac{p(DS,SI)}{p(DS)p(SI)}.$$

We also measured the MI between the strongest synaptic input sequence and somatic spike sequence, i.e., MI (*SS; SI*), where SS represents the occurrence of somatic spikes within the same time bins [somatic spike (*SS*) = 0 for the absence of somatic spike; *SS *=* *1 for the occurrence of somatic spike].

### Code accessibility

Computer simulation was conducted using MATLAB (R2019a, MathWorks Inc.). The MATLAB code that we have generated in this study is freely available online at the public repository GitHub (https://github.com/urakubo/Parajuli).

### Statistics

Statistical tests were performed using IBM SPSS statistics software (IBM, version 24). The normality of the datasets was examined by Shapiro–Wilk test. Student’s *t* test compared statistical significance between two populations for parametric datasets and Mann–Whitney *U* test for data that are distributed in a non-parametric manner. Either one-way ANOVA or the Kruskal–Wallis test was used to examine statistical significance between three or more groups. The correlation was analyzed by Pearson’s correlation or Spearman’s rank order test, as appropriate. Unless otherwise mentioned, data are expressed as mean ± SD. The number of profiles analyzed for statistical analysis is provided in the table. Statistical significance was set at *p *=* *0.05. Single, double and triple asterisks in the figures and tables denote *p* < 0.05, *p* < 0.01, and *p* < 0.001, respectively. Where appropriate, the correlation coefficient and the statistical significance values in the graphs are denoted by *r* and *p*, respectively.

## Results

### Ultrastructure of dendritic spines in multiple brain regions

**Figure 1.**
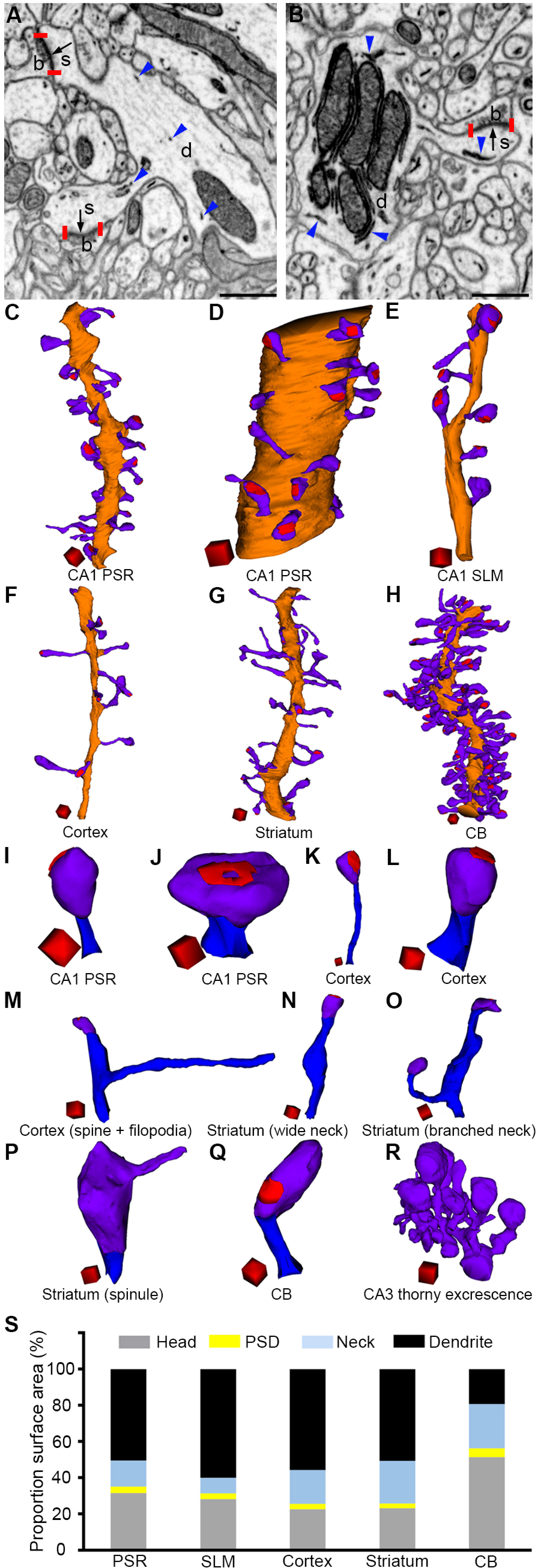
Structural diversity of dendritic spines in the brain. Membrane contours of presynaptic boutons (b), dendrites (d), and spines (s) can be clearly visualized in FIB/SEM images from the cortex (***A***) and CB (***B***). PSDs (indicated by black arrows and delimited by two vertical red bars) and ER (blue arrowheads) are also visible. 3D reconstructions of dendrite (orange), spines (violet), and PSDs (red) in the CA1 PSR (an oblique dendrite, ***C***), CA1 PSR (a large-caliber dendrite, ***D***), CA1 SLM (***E***), cortex (***F***), striatum (***G***), and CB (***H***) show that spines of various morphologies protrude from the same dendrite. Panels ***I–R*** show spines of various morphologies. Panel **J** shows an example of a perforated PSD. Spine heads, spine necks, and PSDs are denoted by violet, blue, and red color, respectively. The bar graph in ***S*** shows the proportion of surface area occupied by spine head, PSD, spine neck, and dendritic shaft. Dendritic spines occupy ∼50% of surface area in the CA1 PSR and striatum, 40% in CA1 SLM, 45% in the cortex, and 80% in CB. Scale bars: 500 nm (***A***, ***B***). Scale cubes: 0.5 μm on each side for all reconstructions except for the CA3 thorny excrescence spine (1 μm on each side).

High-quality, well-preserved morphology is a prerequisite for ultrastructural studies. In our sample, we could visualize plasma membranes, synaptic contacts, and PSDs at a fine level of detail and could resolve the fine morphology of organelles such as mitochondria and endoplasmic reticulum (ER; Fig. 1*A*,*B*). The high-quality images aided unequivocal segmentation of membrane contours. We reconstructed 5- to 25-μm-long dendrites (C-H) by manual segmentation of 150–450 consecutive FIB/SEM images. A total of 86 dendrites and 2078 spines were reconstructed from six different brain regions (CA1 PSR, CA1 SLM, CA3 PSR, layer 1 of the somatosensory cortex, dorsolateral striatum, and the molecular layer of CB). The imaging volume in CA1 PSR and CB were located at a distance of ∼ 100 μm from the edge of the CA1 pyramidal cell layer and Purkinje cell layer, respectively.

Spines of various morphologies were seen protruding from the same parent dendrite. Typically, spines had a narrow neck and bulbous head. However, such a feature was not always apparent, and in some spines, the segregation of head and neck relied on the subjective judgment of the annotator. Thorny excrescence spines in CA3 (Fig. 1*R*) were distinct from the spines in other brain regions as they were unusually large and contained several mitochondria in the spine head. Several spines in CB (Fig. 1*Q*) had a ladle-shaped morphology such that the spine head was tilted roughly at an angle of 60° relative to the spine neck. However, in CA1 PSR, CA1 SLM, cortex, and striatum, both the spine head and spine neck often resided in the same plane (Fig. 1*I–P*). Almost all the spiny protrusions displayed synaptic contacts with their presynaptic partners (Table 1). In the case of CA1 PSR, CA1 SLM, cortex, and striatum, the synaptic contact was mostly located at the tip of the spine head (Fig. 1*I–P*), but it could reside in any part of the spine head in CB (Fig. 1*Q*). PSD in CB was always of macular type, whereas in CA1 SLM, half of the spines had perforated PSD. Less than 10% of the spines in other brain regions had perforated PSD (Table 1). While the spine necks in CA1, cortex, and CB appeared to be cylindrical with roughly similar diameter throughout, striatal spine necks occasionally displayed widening roughly at the midway (Fig. 1*N*). Approximately 10% of the spines were branched in CA1 PSR, cortex, striatum, and CB (Table 1). Sometimes filopodia-like protrusions also branched out from the necks of spines (Fig. 1*M*). Interestingly, we did not encounter any branched spines in CA1 SLM. The proportion surface area occupied by the dendritic shaft, spine head, PSD, and spine neck also varied depending on the brain regions (Fig. 1*S*).

**Table 1:** Frequency of filopodia, perforated spines, spinules, stubby spines, and branched spines in different brain regions

Brain region	Filopodia (%)	Perforated spines (%)	Spinules (%)	Stubby spines (%)	Branched spines (%)	Number of spines in branches (range)
CA1 PSR	1.78	2.72	3.26	2.90	7.97	2
CA1 SLM	0.00	50.00	8.57	1.43	0.00	– –
Cortex	3.07	8.60	0.90	2.26	9.50	2–4
Striatum	5.84	4.47	4.71	0.00	13.40	2–3
CB	4.19	0.00	0.61	0.00	12.62	2–5

Filopodia lack synaptic contacts with the presynaptic terminals.

**Figure 2.**
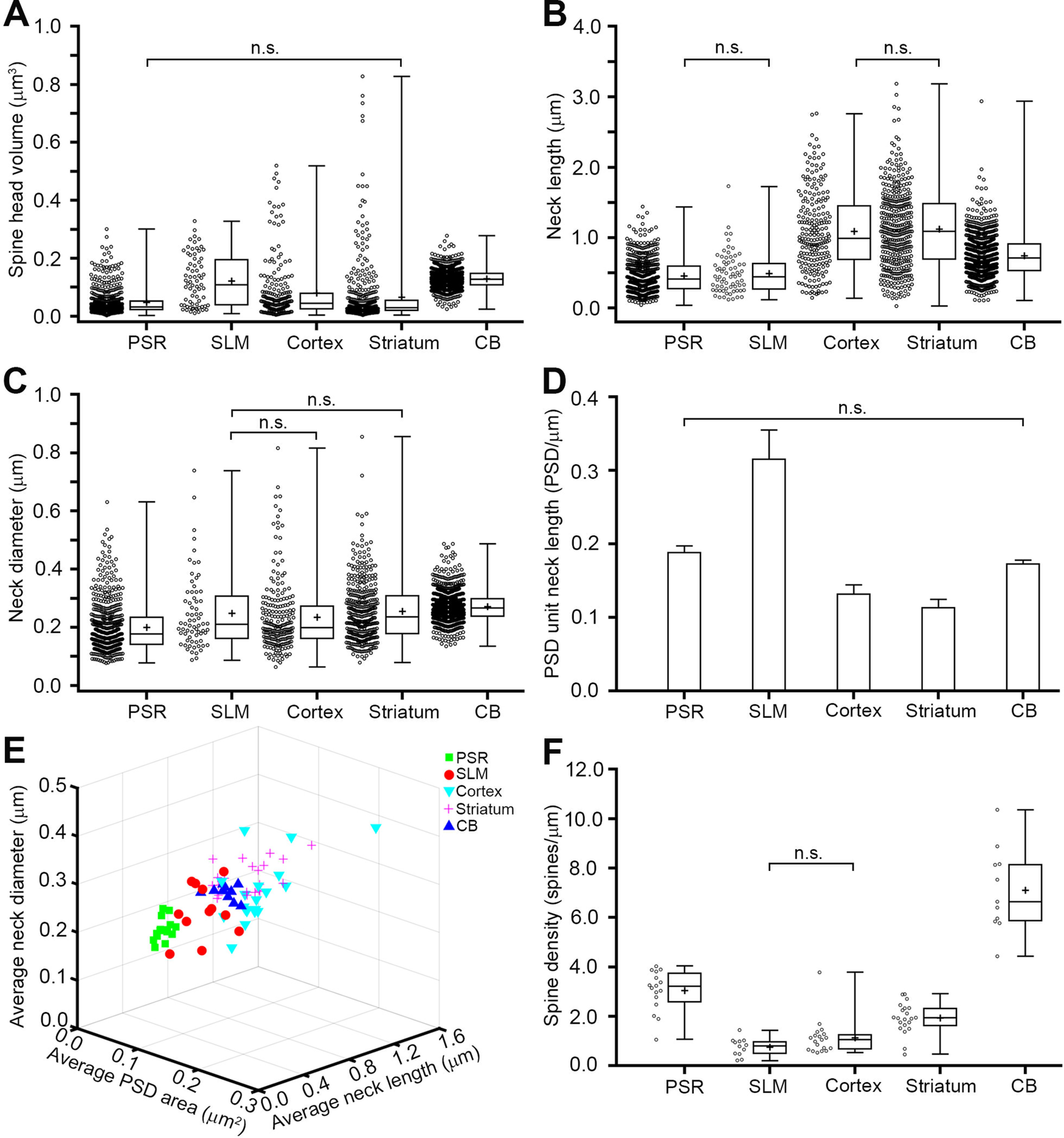
Morphometric analysis of dendritic spines in multiple brain regions. Box and whiskers plots show that the spine head volume (***A***), spine neck length (***B***), and spine neck diameter (***C***) are highly variable, and there exists a significant overlap in their dimensions across different brain regions. Note that, compared with other brain regions, the dendritic spines in CB display much less variability in spine head volume. ***D***, The bar graph shows that the ratio of PSD area to neck length is not significantly different between spines in the CA1 PSR and CB. Error bars indicate SEM. ***E***, 3D plot shows that the dendrites from CA1 PSR, CA1 SLM, cortex, striatum, and CB are not easily distinguishable from each other based on the analysis of the average neck diameter, average PSD area, and average neck length of their spines. ***F***, Box and whiskers plot shows that the spine density is high in the CB, moderate in the CA1 PSR and striatum, and low in the CA1 SLM and cortex. Mean values are indicated by + mark in ***A–C***, ***F***. The box shows 25th, 50th, and 75th percentiles of the dataset. In each graph, a significant difference exists in the spine dimensions between any pair of brain regions not designated as n.s. (not significant). Extended Data Figure 2-1 shows that the spine head volume correlates strongly with the PSD area, but only weakly with the neck length. A linear positive correlation between the amplitude of uEPSC and the spine head volume is shown in Extended Data Figure 2-2. A scatter plot in Extended Data Figure 2-3 shows that the dendrites in the CA1 PSR can be grouped in two distinct populations based on their dendritic diameter.

Next, we analyzed whether each brain region contained spines of distinct dimensions (Fig. 2*A–C*). The mean values for the head volume, neck length, and neck diameter in each brain region are shown in Table 2. The average spine head volumes (in μm^3^) were 0.05 ± 0.045 in CA1 PSR, 0.12 ± 0.086 in CA1 SLM, 0.08 ± 0.101 in cortex, 0.07 ± 0.109 in striatum and 0.13 ± 0.035 in CB. The distribution of head volume was not significantly different between the spines in CA1 PSR and striatum (*p *=* *1.00, Kruskal–Wallis test with Bonferroni correction). However, except for this pair, statistical significance could be detected when spine head volume was compared between any of the two regions studied here (*p* values between CA1 PSR and CA1 SLM < 0.001, CA1 PSR and cortex < 0.01, CA1 PSR and CB < 0.001, CA1 SLM and cortex < 0.001, CA1 SLM and striatum < 0.001, CA1 SLM and CB = 0.03, cortex and striatum = 0.01, cortex and CB < 0.001, striatum and CB < 0.001, Kruskal–Wallis test with Bonferroni correction). Despite the significant differences in the spine head volume, spines from different regions of the brain were not readily distinguishable from each other because of the significant overlap in their head dimensions (Fig. 2*A*). There was a 124- to 204-fold difference in the head volume dimensions between the smallest and the largest spines in CA1 PSR, cortex, and striatum. Relatively smaller variation was observed in CA1 SLM (35-fold) and CB (11-fold).

**Table 2:** Dimensions of spine head and spine neck in different brain regions

Brain region	Spines Dendrites (*n*)	Spine density (mean ± SD)	Head volume (mean ± SD, ratio, CV)	Neck length (mean ± SD, ratio, CV)	Neck diameter (mean ± SD)	Ratio of PSD area to neck length (mean ± SD)
CA1 PSR	552 23	3.04 ± 0.825	0.05 ± 0.045 124, 0.94	0.46 ± 0.246 38, 0.54	0.20 ± 0.087	0.19 ± 0.193
CA1 SLM	70 12	0.75 ± 0.360	0.12 ± 0.086 35, 0.71	0.49 ± 0.289 15, 0.59	0.25 ± 0.127	0.32 ± 0.323
CA3 PSR	8 —	—	1.66 ± 1.988 155, 1.20	—	—	—
Cortex	221 19	1.14 ± 0.723	0.08 ± 0.101 159, 1.26	1.09 ± 0.561 20, 0.52	0.23 ± 0.121	0.13 ± 0.169
Striatum	403 21	1.94 ± 0.621	0.07 ± 0.109 204, 1.66	1.12 ± 0.556 106, 0.50	0.26 ± 0.104	0.11 ± 0.204
CB	824 11	7.10 ± 1.693	0.13 ± 0.035 11, 0.27	0.74 ± 0.300 27, 0.40	0.27 ± 0.054	0.17 ± 0.130

Units: spine density, spines/μm; head volume, μm^3^; neck length, μm; neck diameter, μm.

Only the small-diameter dendrites (diameter < 1 μm) are included for the spine density analysis in the CA1 PSR. Out of 23 dendrites analyzed, 16 dendrites fulfilled this criterion. All the spines, regardless of the dendritic diameter of their parent dendrites, are included for head volume, neck length and neck diameter analysis.

Ratio of head volume indicates the fold difference between the largest and smallest spine heads. Ratio of neck length indicates the fold difference between the longest and shortest spine necks. CV, coefficient of variation.

10.1523/ENEURO.0248-20.2020.f2-1 Extended Data Figure 2-1 Spine head volume correlates strongly with the PSD area but only weakly with the neck length. Scatter plots show a significant positive correlation between head volume and PSD area in the CA1 PSR (***A***), CA1 SLM (***B***), cortex (***C***), striatum (***D***), and CB (***E***). Scatter plots show that there is no significant correlation between head volume and neck length in the CA1 PSR (***F***), CA1 SLM (***G***), and cortex (***H***). A significant negative correlation is observed between head volume and neck length in the striatum (***I***) and CB (***J***). Head volume in ***F–J*** is expressed on a logarithmic scale. Download Figure 2-1, TIF file.

10.1523/ENEURO.0248-20.2020.f2-2 Extended Data Figure 2-2 The amplitude of uEPSC positively correlates with the spine head volume. ***A***, Two-photon image of a CA1 pyramidal neuron from an acute hippocampal slice recorded in whole-cell voltage-clamp mode. uEPSCs (blue trace) from target spines were evoked by the application of glutamate uncaging test pulses (7–10 trials at 0.1 Hz) and recorded from the CA1 pyramidal cell soma maintained at a holding potential of –65 mV. ***B***, Two-photon image of a dendritic segment (corresponding to the region shown by the red square in ***A***) showing locations of glutamate uncaging (blue crosses). ***C***, Amplitude of uEPSCs plotted against individual spine volume (open circles, *n* = 140 spines, 33 dendrites, 12 cells). Note that the uEPSC amplitudes recorded at the soma show a significant positive correlation with the estimated spine volume. On average, four spines per dendrite were examined. Download Figure 2-2, TIF file.

**Figure 3.**
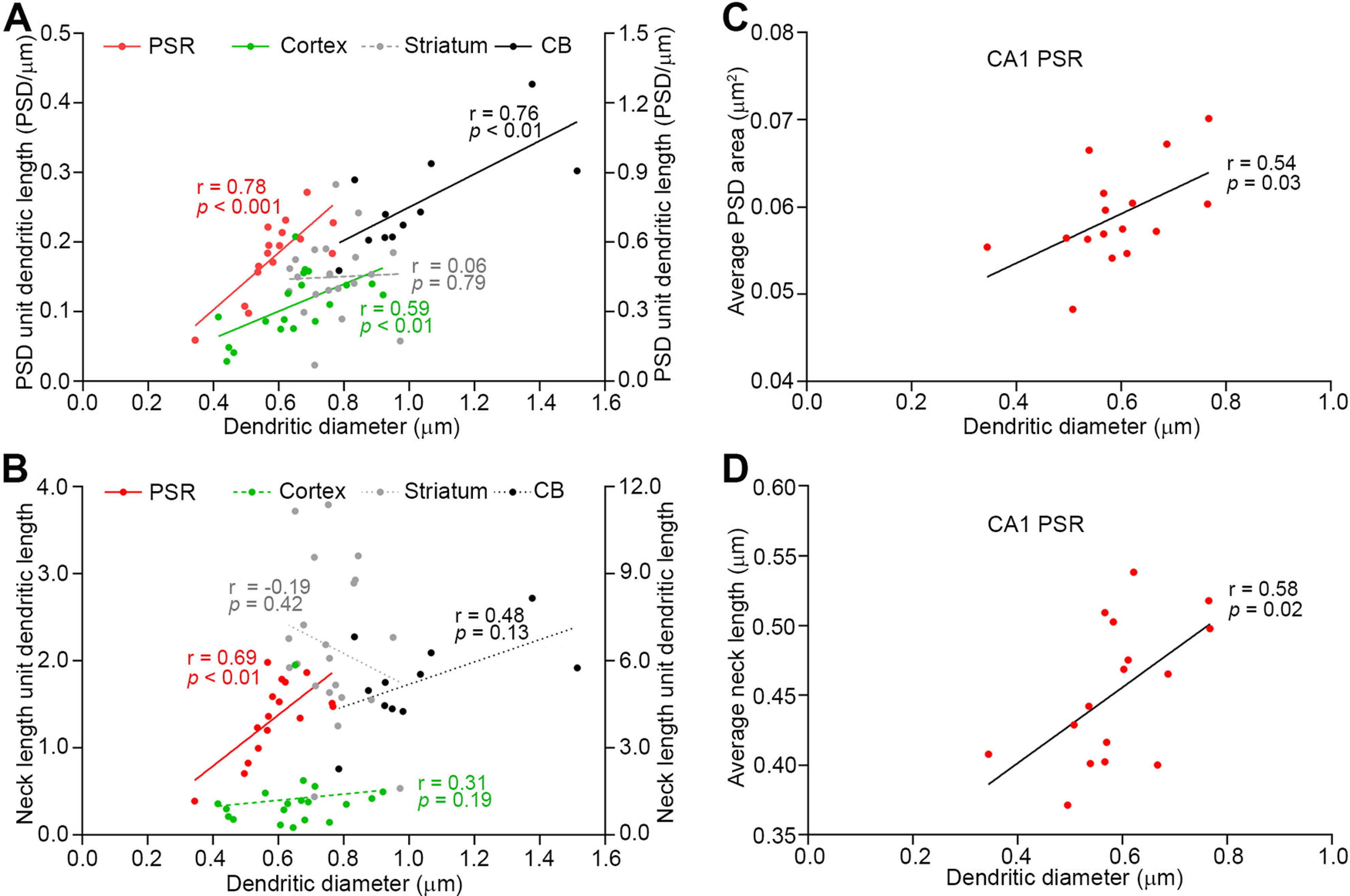
Spines are organized in a regulated manner in the dendrites. ***A***, PSD area unit dendritic length shows a significant positive correlation with the dendritic diameter in the CA1 PSR, cortex, and CB (see also Extended Data Fig. 3-1). However, in the striatum, the PSD area unit dendritic length does not correlate with dendritic diameter. ***B***, Neck length unit dendritic length is positively correlated with the dendritic diameter in the CA1 PSR. The data from CA1 PSR, cortex, and striatum are plotted in reference to the left vertical axis, and the data from CB are plotted in reference to the right vertical axis in panels ***A***, ***B***. Average PSD area (***C***), and average neck length (***D***) show a positive correlation with the dendritic diameter in the CA1 PSR. Scatter plots in Extended Data Figure 3-2 show that the ratio of the total PSD area to total neck length (***A***), and the average neck diameter (***B***) do not correlate with the dendritic diameter in the CA1 PSR. Extended Data Figure 3-3 shows that the synaptic constancy enables effective information transfer from the dendrite to the soma.

10.1523/ENEURO.0248-20.2020.f2-3 Extended Data Figure 2-3 CA1 PSR dendrites can be grouped into two distinct populations based on the dendritic diameter. Dendrites in the CA1 PSR can be grouped into two populations based on their dendritic diameters. Dendrites with a diameter <1 μm likely represent the oblique dendrites. However, the large-diameter dendrites (>1.4 μm) can either be the main apical shafts or the segments of oblique dendrites near to the branch points. Download Figure 2-3, TIF file.

Spine neck dimensions also differed among brain areas (Fig. 2*B*). Despite the significant differences in the distribution of spine head volume, statistical significance was not revealed in the distribution of neck lengths between the spines in CA1 PSR and CA1 SLM, and between the spines in cortex and striatum [average neck lengths (in μm) in CA1 PSR = 0.46 ± 0.246, CA1 SLM = 0.49 ± 0.289, cortex = 1.09 ± 0.561, striatum = 1.12 ± 0.556, CB = 0.74 ± 0.300; *p* values between CA1 PSR and CA1 SLM = 1.00, cortex and striatum = 1.00, Kruskal–Wallis test with Bonferroni correction]. Except for these pairs, all other brain regions showed statistical significance in the distribution of spine neck length (*p* values between CA1 PSR and cortex < 0.001, CA1 PSR and striatum < 0.001, CA1 PSR and CB < 0.001, CA1 SLM and cortex < 0.001, CA1 SLM and striatum < 0.001, CA1 SLM and CB < 0.001, cortex and CB < 0.001, striatum and CB < 0.001; Kruskal–Wallis test with Bonferroni correction). Similar to the large variability observed in the head volume, the ratio of neck lengths of the longest to the shortest necks also varied by several fold (fold differences: CA1 PSR = 38, CA1 SLM = 15, cortex = 20, striatum = 106, CB = 27; Table 2).

Next, we tested whether the brain regions with longer spine necks also showed the tendency to have spines with wide necks. However, we found that the spine neck length is a poor predictor of the spine neck diameter (Fig. 2*C*; Table 2). In particular, spines in cortex and striatum had longer necks than the spines in other brain regions. However, cortex and striatum neither had the widest, nor the narrowest, neck diameter [average neck diameter (in μm) in CA1 PSR = 0.20 ± 0.087, CA1 SLM = 0.25 ± 0.127, cortex = 0.23 ± 0.121, striatum = 0.26 ± 0.104, CB = 0.27 ± 0.054]. Cortical and striatal spines had significantly thicker necks than the spines in CA1 PSR and significantly thinner necks than the spines in CB (*p* values between CA1 PSR and cortex < 0.01, CA1 PSR and striatum < 0.001, cortex and CB < 0.001, striatum and CB < 0.001, Kruskal–Wallis test with Bonferroni correction). Spine neck diameter in CA1 PSR was significantly smaller than in the CB (*p* value between CA1 PSR and CB < 0.001, Kruskal–Wallis test with Bonferroni correction). The distribution of spine neck diameter in cortex and striatum was not significantly different from the spines in CA1 SLM (*p* values between CA1 SLM and cortex = 1.00, CA1 SLM and striatum = 1.00, Kruskal–Wallis test with Bonferroni correction). In contrast, CA1 SLM spines had significantly wider necks compared with CA1 PSR and significantly narrower neck compared with CB (*p* values between CA1 SLM and CA1 PSR < 0.01, CA1 SLM and CB < 0.001, Kruskal–Wallis test with Bonferroni correction). Interestingly, despite the similarity in the neck lengths, a significant difference in neck diameter was observed between the spines in cortex and striatum (*p* value between cortex and striatum < 0.01, Kruskal–Wallis test with Bonferroni correction).

**Figure 4.**
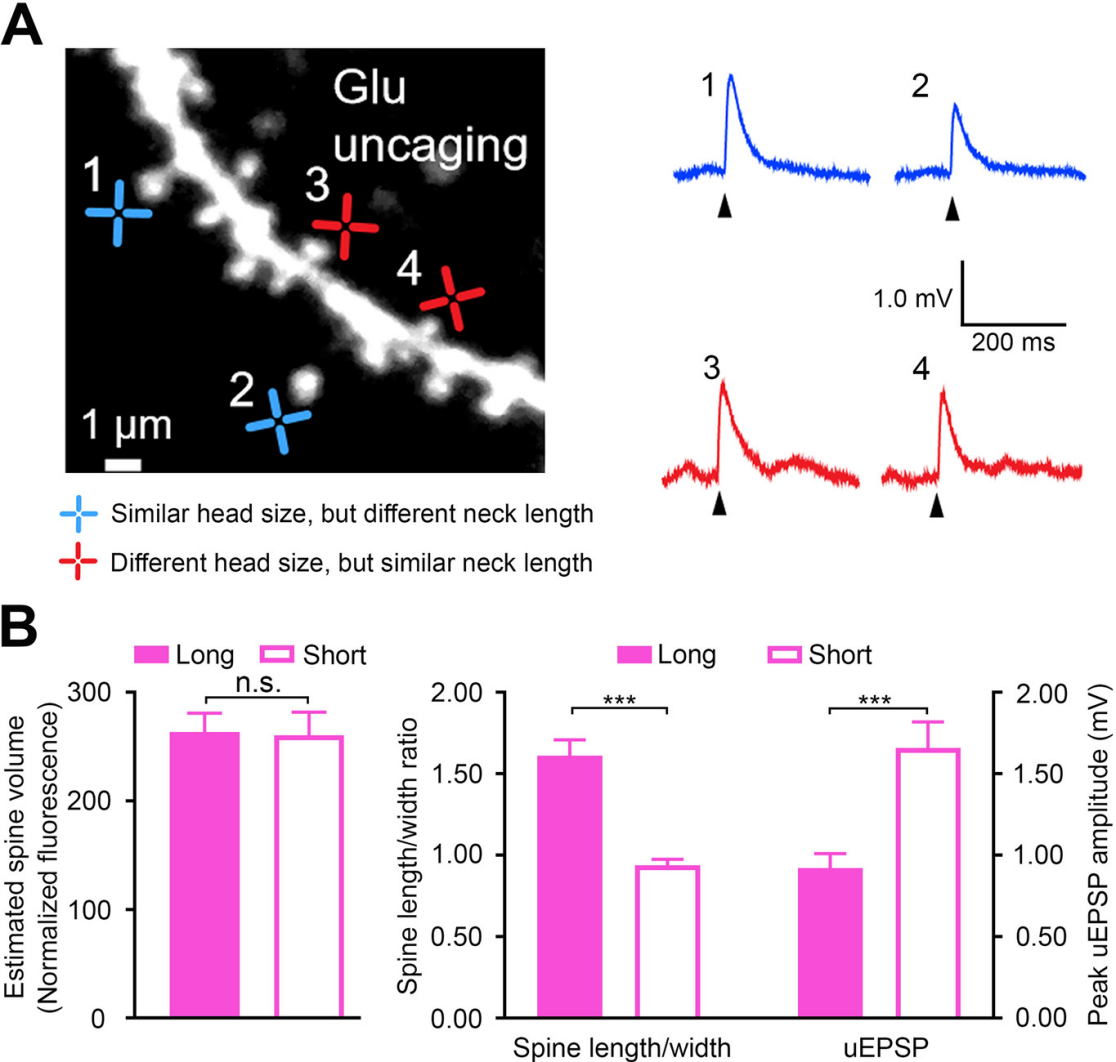
Spine neck strongly filters membrane potential on CA1 pyramidal neurons. ***A***, Two-photon image of a dendritic segment showing the spines (blue crosses: spines of similar head size, but different neck length; red crosses: spines of different head size, but similar neck length) receiving glutamate uncaging stimulus. uEPSPs were evoked by glutamate uncaging test pulses (5–10 trials at 0.1 Hz). Blue and red traces are representative uEPSPs from blue and red targets, respectively. ***B***, Quantitative analyses of the estimated spine volume, spine length to width ratio, and uEPSPs of long (filled bars) and short (open bars) neck spines (*n* = 35 spines in each group, 6 cells). Error bars indicate SEM; n.s., not significant. See Extended Data Figure 4-1 for the relationship of the uEPSP amplitude with the spine neck length and the head width.

The magnitude of EPSPs at the soma, generated by the activation of a dendritic spine, is critically influenced by the dimensions of the spine head volume and the neck length (Matsuzaki et al., 2001; Araya et al., 2006b, 2014). The spine head volume is positively correlated with the PSD area (CA1 PSR, *r* = 0.80, *p *<* *0.001; CA1 SLM, *r* = 0.89, *p *<* *0.001; cortex, *r* = 0.86, *p *<* *0.001; striatum, *r* = 0.80, *p *<* *0.001; CB, *r* = 0.42, *p *<* *0.001; Spearman’s rank order test; Extended Data Fig. 2-1*A–E*) and, in turn, the PSD area positively correlates with the AMPA receptor content (Nusser et al., 1998). This suggests that the size of the spine head is an important parameter that determines the magnitude of synaptic strength. Indeed, using two-photon microscopy, glutamate uncaging and electrophysiological recordings in hippocampal CA1 pyramidal neurons, we revealed that the magnitude of uEPSC recorded at the soma is positively correlated with the head volume of the activated spines (Extended Data Fig. 2-2). In contrast to the role of spine head in somatic membrane depolarization, spine necks attenuate membrane potentials (Araya et al., 2006b, 2014; Fig. 4; Extended Data Fig. 4-1). Thus, larger spine heads (i.e., larger PSD areas) and shorter spine necks generate stronger membrane depolarization at the soma than the spines with smaller heads and longer necks. Simply put, the synaptic strength of a spine can be expressed by the ratio of the spine head volume (or the PSD area) to the spine neck length. Interestingly, despite the large differences in the average head volume and the average neck length of spines between CA1 PSR and CB, we found that the ratio of PSD area to neck length was similar between these two regions (CA1 PSR: 0.19 ± 0.193, CB: 0.17 ± 0.130; *p *=* *1.00, Kruskal–Wallis test with Bonferroni correction; Fig. 2*D*). However, the spines in other brain regions were significantly different in terms of the ratio of the PSD area to the neck length (CA1 SLM: 0.32 ± 0.323, cortex: 0.13 ± 0.169, striatum: 0.11 ± .204; *p* values between CA1 PSR and CA1 SLM < 0.01, CA1 PSR and cortex < 0.001, CA1 PSR and striatum < 0.001, CA1 SLM and cortex < 0.001, CA1 SLM and striatum < 0.001, CA1 SLM and CB = 0.01, cortex and striatum < 0.01, cortex and CB < 0.001, striatum and CB < 0.001; Kruskal–Wallis test with Bonferroni correction; Fig. 2*D*). Head volume and neck length are likely to be independently regulated as either no correlation or only a weak, but statistically significant, correlation was observed between these two parameters (CA1 PSR: *r* = 0.02, *p *=* *0.63; CA1 SLM: *r* = −0.11, *p *=* *0.38; cortex: *r* = −0.12, *p *=* *0.08; striatum: *r* = −0.27, *p *<* *0.001; CB: *r* = −0.15, *p *<* *0.001; Spearman’s rank order test; Extended Data Fig. 2-1*F–J*).

After having obtained the precise measurement of the spine head volume, spine neck length, and spine neck diameter, we asked whether the combination of these structural features of spines would enable us to identify the brain region where the spines were sampled. The average neck length, average PSD area, and average neck diameter of spines was obtained from each dendrite and plotted in *x*-, *y*-, and *z*-axes. Since it is not possible to segregate individual spines from different brain regions because of large variability in their dimensions (Fig. 2*A–C*), we wondered whether averaging the dimensions of spines in each dendrite would make it possible to identify the region that a dendrite is sampled from. However, a 3D scatter plot revealed that the dendrites from different brain regions show a considerable overlap in the average spine dimensions (Fig. 2*E*). We conclude that based on the combination of average neck length, average PSD area, and average neck diameter, it is not possible to unequivocally identify the sampled brain region.

The relative abundance of dendritic spines differs between the brain regions (Fig. 2*F*). We calculated the density of spines in individual dendrites by dividing the total number of spines by the length of the dendrite. The Purkinje cell dendrites had the highest density of spines, followed by the dendrites in CA1 PSR and striatum [spine density (spines/μm) in CB = 7.10 ± 1.693, CA1 PSR = 3.04 ± 0.825, striatum = 1.94 ± 0.621]. Dendritic spines were sparse in the dendrites of CA1 SLM and cortex [spine density (spines/μm) in CA1 SLM = 0.75 ± 0.360, cortex = 1.14 ± 0.723] and the spine density was not significantly different between these two regions (*p *=* *0.45, one-way ANOVA). However, except for this pair, the difference in spine density was statistically significant between any of the two regions studied here (*p* values between CA1 PSR and CA1 SLM < 0.001, CA1 PSR and cortex < 0.001, CA1 PSR and striatum < 0.01, CA1 PSR and CB < 0.001, CA1 SLM and striatum < 0.001, CA1 SLM and CB < 0.001, cortex and striatum <0.01, cortex and CB < 0.001, striatum and CB < 0.001, one-way ANOVA).

Our data thus far revealed that the dimensions of spines and their relative abundance differ according to the brain regions. Next, we calculated the average PSD area unit dendritic length and the average neck length unit dendritic length in each brain region (Table 3). These values are influenced by both the intrinsic structural properties of spines (PSD size or neck length) and the spine density. We observed that the average PSD area unit dendritic length is highest in the CB, followed by that in the CA1 PSR, striatum, cortex, and CA1 SLM (CA1 PSR: 0.18 ± 0.055, CA1 SLM: 0.08 ± 0.031, cortex: 0.11 ± 0.046, striatum: 0.15 ± 0.057, CB: 0.77 ± 0.221). Similarly, the average neck length unit dendritic length is highest in the CB, followed by that in the striatum, CA1 PSR, cortex, and CA1 SLM (CA1 PSR: 1.35 ± 0.442, CA1 SLM: 0.37 ± 0.268, cortex: 1.25 ± 1.205, striatum: 2.16 ± 0.906, CB: 5.29 ± 1.539). Furthermore, the ratio of the summed PSD area to the summed neck length averaged from each dendrite was also similar between CA1 PSR and CB (Table 3).

**Table 3:** PSD area unit dendritic length and the neck length unit dendritic length in different brain regions

Brain region	Dendritic diameter (mean ± SD)	PSD area unit dendritic length (mean ± SD)	Neck length unit dendritic length (mean ± SD)	Ratio of total PSD area to total neck length in dendrites (mean ± SD)
CA1 PSR	0.59 ± 0.104	0.18 ± 0.055	1.35 ± 0.442	0.14 ± 0.018
CA1 SLM	0.58 ± 0.097	0.08 ± 0.031	0.37 ± 0.268	0.27 ± 0.112
Cortex	0.65 ± 0.141	0.11 ± 0.046	1.25 ± 1.205	0.12 ± 0.082
Striatum	0.77 ± 0.096	0.15 ± 0.057	2.16 ± 0.906	0.08 ± 0.029
CB	1.02 ± 0.226	0.77 ± 0.221	5.29 ± 1.539	0.15 ± 0.024

Units: dendritic diameter, μm; PSD area unit dendritic length, μm; PSD area unit neck length, μm.

### Organizational principle of dendritic spines along the dendrites in different brain regions

It is of great interest to reveal how the excitatory synaptic inputs are organized along a dendritic arbor. Dendrites actively integrate synaptic weights and serve as an independent computational unit (Stuart and Spruston, 2015). Dendrites are roughly cylindrical structures whose diameter progressively decreases toward the distal tips (Hillman, 1979; Larkman et al., 1992). Small-diameter dendrites have high input resistance, and consequently, a small number of inputs are sufficient to evoke dendritic action potentials. In contrast, in the case of large-diameter dendrites, a large number of inputs are necessary to achieve the membrane depolarization high enough to reach the threshold for dendritic action potential initiation. We selected PSD area density (summation of multiple PSD areas along a dendrite divided by the dendritic length) as a measure of synaptic strength, instead of spine density. This selection of the PSD-based parameter is supported by the notion that the PSD area correlates with the functional synaptic strength (Nusser et al., 1998; Matsuzaki et al., 2001). Spine density measurement assigns equal synaptic weights to each spine regardless of their sizes. However, both the PSD area and the spine size are highly variable and mutually correlated (Extended Data Fig. 2-1*A–E*). As such, in comparison to the spine with the small PSD area, it is more reasonable to assign higher synaptic weights to the spine with the large PSD area. Thus, in comparison to the spine density, PSD area density is a better estimate of the synaptic strength of dendrites.

The spiny regions that we have studied differ in terms of microanatomy and cellular topography. We first limited our analysis of the relationship between dendritic diameter and PSD area per unit length of dendrite to CA1 PSR and CB. In both regions, somata of the principal neurons are confined to a specific layer. Therefore, dendrites sampled at the neuropil ∼100 μm away from the edge of the cell layer can be anticipated to be relatively homogeneous in terms of their structure and function. However, contrary to our expectations, dendrites in CA1 PSR could be divided into two distinct populations based on the dendritic diameter (Extended Data Fig. 2-3). Dendrites with a diameter of <1 μm were categorized as one group and those with a diameter larger than 1.4 μm as another group. Since this second population (dendritic diameter >1.4 μm) is highly likely to be contaminated by the dendrites of the main apical trunk, which are known to have very few to no spines, we limited our analysis to the first group (i.e., dendritic diameter <1 μm). This group of dendrites further demonstrated heterogeneity in the dendritic diameter, which may serve as a putative indicator of the distance of the dendrites from the dendritic branch points. A positive correlation was observed between dendritic diameter and total PSD area per unit length of dendrite in both CA1 PSR and CB (CA1 PSR: *r* = 0.78, *p *<* *0.001, Pearson’s correlation; CB: *r* = 0.76, *p *<* *0.01, Spearman’s rank order test; Fig. 3*A*). Next, the layered organization of the cortex prompted us to examine whether the statistically significant correlation between dendritic diameter and total PSD area per unit length of a dendrite, observed in CA1 PSR and CB, can also be found in the cortex. Although the cell bodies in the neocortex form discrete layers, the dendrites sampled in layer 1 could belong to cells in any of the underlying layers. Thus, because of the variation in the cell-type, the dendrites sampled from layer 1 are likely to be electrically heterogeneous in terms of their distance from the soma. However, despite this heterogeneity, we found that the PSD area per unit length of dendrite correlates with the dendritic diameter in the cortex (*r* = 0.59, *p *<* *0.01, Pearson’s correlation; Fig. 3*A*). In the case of the striatum, the cell body is not confined to a specific layer. Therefore, the relationship between the summed PSD areas and the relative position along dendrites is difficult to correlate. In other words, a given imaged region of the striatum is highly likely to contain dendrites that are located at varying distances from their parent soma. In the case of CA1 SLM, the presence of shaft excitatory synapses in their dendrites makes the calculation of total anatomic strength ambiguous. It has been previously shown that the inputs in dendritic shaft summate in a sublinear manner (Araya et al., 2006a), which is different compared with the linear integration of excitatory inputs on spines. Likely attributable to these reasons, no correlation between dendritic diameter and PSD area was observed in the striatum (*r* = 0.06, *p *=* *0.79, Pearson’s correlation; Fig. 3*A*) and CA1 SLM (*r* = 0.26, *p *=* *0.42, Pearson’s correlation; data not shown). Summation of PSD areas both from the spine head and dendritic shaft in CA1 SLM also did not result in any significant correlation with the dendritic diameter (*r* = 0.44, *p *=* *0.15, Pearson’s correlation). Nevertheless, our data demonstrate that the dendritic diameter serves as a useful indicator of the PSD content in CA1 PSR, cortex, and CB. Moreover, the result from striatum and CA1 SLM also argues that the higher PSD content in large-diameter dendrites is not simply because of the availability of the larger surface area for synaptic contacts.

Spine neck is another important compartment of a dendritic spine that governs the magnitude of the synaptic strength. As observed for the total PSD area in a dendrite, the total neck length also scaled positively with a dendritic diameter in CA1 PSR (CA1 PSR, *r* = 0.69, *p *<* *0.01, Pearson’s correlation; Fig. 3*B*). In cortex and CB there was a trend for an increase in total neck length per unit dendritic length with the dendritic diameter, but the correlation was not statistically significant (cortex, *r* = 0.31, *p *=* *0.19; CB, *r* = 0.48, *p *=* *0.13; Spearman’s rank order test; Fig. 3*B*).

Next, we examined whether the statistically significant positive correlation in Figure 3*A*,*B* is because of the higher spine density in large-diameter dendrites. The spine density positively correlates with the dendritic diameter in CA1 PSR (*r* = 0.71, *p *<* *0.01, Pearson’s correlation; Extended Data Fig. 3-1*A*). However, although there was a trend toward higher spine density in the large-diameter dendrites in cortex and CB, the data did not reach statistical significance (cortex, *r* = 0.43, *p *=* *0.07; CB, *r* = 0.47, *p *=* *0.14; Spearman’s rank order test; Extended Data Fig. 3-1*B*,*C*). As noted earlier, the PSD area differs depending on the spine size, and as such, the contribution of each spine to the total synaptic strength is not equal. As a result, the plot of spine density versus dendritic diameter and the PSD area density versus dendritic diameter may not necessarily align with each other. It is reasonable to assume that both the spine density and the size of PSD contribute to the correlation between PSD area density and the dendritic diameter in multiple brain regions. The contribution of these two factors is clearly illustrated in the case of CA1 PSR, where both the spine density (Extended Data Fig. 3-1*A*) and the average PSD area of spines (*r* = 0.54, *p *=* *0.03, Pearson’s correlation; Fig. 3*C*) show a significant positive correlation with dendritic diameter. Additionally, the average neck length is also positively correlated with the dendritic diameter in CA1 PSR (*r* = 0.58, *p *=* *0.02, Pearson’s correlation; Fig. 3*D*). Taken together, we propose that the intrinsic structural properties of spines differ across different dendrites, and both the spine density and the individual PSD size contribute to the higher PSD content unit length of dendrite in a large-diameter dendritic segment in CA1 PSR, cortex and CB.

10.1523/ENEURO.0248-20.2020.f3-1 Extended Data Figure 3-1 Spine density is positively correlated with the dendritic diameter in the CA1 PSR. Scatter plots of spine density against dendritic diameter in the CA1 PSR (***A***), cortex (***B***), and CB (***C***). A significant positive correlation is detected between the spine density and the dendritic diameter in the CA1 PSR. Download Figure 3-1, TIF file.

The data thus far showed that the four parameters (the summed PSD area unit dendritic length, average PSD area, summed neck length unit dendritic length, and average neck length) increase with the dendritic diameter in CA1 PSR. As mentioned previously, the ratio of the PSD area to the spine neck length may be a useful index for the estimation of the synaptic strength of a spine. Since both the PSD area and the spine neck length show a positive correlation with the dendritic diameter, the ratio between these two parameters can be assumed to remain relatively similar across different dendrites. Experimentally, this hypothesis was confirmed to be true in CA1 PSR (Extended Data Fig. 3-2*A*). Besides, the neck diameter (another important structural parameter that influences the synaptic strength of a dendritic spine) also does not show an apparent correlation with the dendritic diameter (Extended Data Fig. 3-2*B*). These facts suggest that, despite the positive relationship of the spine head volume and the neck length with the dendritic diameter, synaptic strength may not vary systematically among dendrites of different diameters in the CA1 PSR.

10.1523/ENEURO.0248-20.2020.f3-2 Extended Data Figure 3-2 Neither the ratio of PSD area to neck length, nor the average neck diameter correlates with the dendritic diameter in the CA1 PSR. Scatter plots show that neither the ratio of total PSD area to total neck length (***A***), nor the average neck diameter (***B***) correlates with the dendritic diameter in the CA1 PSR. Download Figure 3-2, TIF file.

10.1523/ENEURO.0248-20.2020.f3-3 Extended Data Figure 3-3 Synaptic constancy enables effective information transfer from the dendrite to the soma. ***A***, Schematic representation of a two-layer neuron model. A dendritic branch receives inputs from 250 different synapses. Dendritic spikes from twenty branches were integrated at the soma for somatic spiking. ***B***, Case 1 shows that the synaptic strength in all the branches obeys single lognormal distribution, and thus, the dendrites are of similar synaptic strength. Case 2 shows that the synaptic strength in each branch obeys a lognormal distribution, but the mean synaptic strengths (*μ_e_*) differs across branches. The mean *μ_e_* was chosen from the Gaussian distribution *N*(−6, 0.4). ***C***, MI between the synaptic inputs and the occurrence of dendritic spikes. The synaptic input from the strongest synapse was compared with the characteristics of the resultant dendritic spike. ***D***, MI between the synaptic inputs and somatic spikes. ***C***, ***D***, Left MI plots are based on the data from case 1, and the right MI plots are based on the data from case 2. Download Figure 3-3, TIF file.

### Analysis of information transfer in a model CA1 neuron

Since the ratio of PSD area to neck length was relatively similar across dendrites of different diameters, we were motivated to clarify the physiological relevance of synaptic constancy across dendrites in CA1 PSR. To address this, we developed an abstract two-layer model of a CA1 neuron (Extended Data Fig. 3-3). In this model, excitatory synaptic inputs cause an increase in membrane potentials in their parent dendritic branches and result in the firing of dendritic spikes if the membrane potentials exceed a certain threshold (Häusser and Mel, 2003; Barnes et al., 2017). Such dendritic spikes were then converged and processed for somatic spiking. In our model, we assumed two different scenarios of synaptic strength distribution in dendrites (cases 1 and 2). In case 1, all synaptic strengths obey an identical lognormal distribution (Extended Data Fig. 3-3*B*, left). In case 2, synaptic strengths at any particular branch obey a lognormal distribution, but its location parameter (*μ_e_*, the median of the lognormal distribution) differs across branches (Extended Data Fig. 3-3*B*, right). Synaptic strengths in case 1 have uniform median values, but those in case 2 do not. We then simulated the dendritic and somatic spikes of the two-layer model neuron in response to Poisson inputs to all the synapses. Next, we targeted the input sequence only to the strongest synapse of each branch, and the relationship between the synaptic input to the strongest synapse and the occurrence of the dendritic spike was studied by MI (Extended Data Fig. 3-3*C*). MI showed a specific tuning curve as a function of synaptic input frequency (Extended Data Fig. 3-3*C*, left). At low input frequency, dendritic branches could not elicit dendritic spikes. Hence no information was transmitted (MI = 0). It was observed that at moderate input frequency, a large number of weak synaptic inputs effectively supported the occurrence of dendritic spike initiated by the strongest synaptic input, thereby causing an increase in the MI transfer. However, the higher frequency inputs disturbed the neuronal information transmission from the dendrites toward the soma. Similar to our findings, Teramae et al. (2012) have also previously reported that a moderate level of noise enhances the information transfer of a nonlinear system driven by the weak and aperiodic input. Furthermore, the magnitude of synaptic strength in the dendrite also clearly influenced the synaptic input frequency-dependent tuning curve (Extended Data Fig. 3-3*C*, right). Particularly, lowering the synaptic strength in a dendrite increased the input frequency value required for achieving an optimal MI [compare the input frequency values required for lower (*μ_e_* = −6.4) and higher (*μ_e_* = −5.6) synaptic strengths]. This result shows that there exists an optimal frequency value for each distinct synaptic strength. Consequently, if synaptic strengths in all the branches obey a single lognormal distribution, the MI between the synaptic inputs and the somatic spikes is highly tuned to a single input frequency (Extended Data Fig. 3-3*D*, left). In contrast, MI value decreases when the synaptic strength differs across branches (Extended Data Fig. 3-3*D*, right). These results suggest that the constancy in synaptic strength across branches enables the effective transfer of information from the strongest synaptic input to the soma.

### Relative role of spine head and neck in EPSPs at the soma

The observation that both the PSD area and neck length correlates positively with a dendritic diameter in CA1 PSR is particularly interesting. These two structural components of spines have an opposing influence on the voltage change at the dendrite. Whereas the larger PSD area leads to a higher current influx into the dendrite, the longer necks cause a significant attenuation of membrane potentials because of the electrical resistance of the neck (Araya et al., 2006b, 2014). If we simply overlook the influence of spine necks, the positive correlation between summed PSD area and dendritic diameter suggests higher synaptic strength in large dendrites. However, it is also equally possible that voltage attenuation caused by the spine neck supersedes the depolarizing influence of the spine head. In this scenario, as a result of the differences in the neck lengths, the spines located in the small-diameter dendrites are expected to exert a considerably stronger influence on the dendritic membrane potential changes than the spines located in the large-diameter dendrites.

To assess the relative role of the spine head and spine neck in EPSPs, we measured uEPSPs from individual spines (Fig. 4*A*) and examined the uEPSPs recorded at the soma against neck lengths and spine head widths (Extended Data Fig. 4-1). As previously reported in the cortex (Araya et al., 2006b, 2014), the peak uEPSP amplitudes were negatively correlated with the spine neck lengths (*r* = −0.33, *p *<* *0.01; Extended Data Fig. 4-1*A*,*B*), but not with the head widths (*r* = −0.07, *p *=* *0.57, Spearman’s rank order test; Extended Data Fig. 4-1*C*). To determine the role of spine neck in EPSPs at a single spine level, we divided spines that had similar head volumes (*p *=* *0.60; Mann–Whitney *U* test; Fig. 4*B*) into two groups based on spine length to head width ratio (Woods et al., 2011). The ratio of spine length to head width was significantly larger in long neck spine groups compared with the short neck spines group (ratio in long neck spine groups = 1.61 ± 0.586, short neck spine groups = 0.94 ± 0.208, *p *<* *0.001, Mann–Whitney *U* test; Fig. 4*B*). We found that the uEPSPs were significantly smaller in the long neck spines than in the short neck spines (long neck spines group = 0.92 ± 0.54 mV, short neck spines group = 1.66 ± 0.94 mV, *p *<* *0.001, Mann–Whitney *U* test; Fig. 4*B*). These data strongly suggest that neck length has a more significant influence than spine head size on voltage changes at the soma.

10.1523/ENEURO.0248-20.2020.f4-1 Extended Data Figure 4-1 The amplitudes of uEPSPs negatively correlate with the spine neck lengths. ***A***, ***B***, Plots of the uncaging potentials (peak amplitude) versus spine neck lengths. The same plot in ***A*** is plotted in ***B*** by assigning a value of 0.2 μm to all the neck lengths shorter than 0.2 μm. The red circle indicates the average EPSPs of the spines whose neck lengths were shorter than 0.2 μm. C, Plot of the uncaging potentials (peak amplitude) versus head widths. Each circle in ***A–C*** represents dendritic spines (*n* = 70 spines, 22 dendrites, 6 cells). Lines are linear fit (Spearman’s rank order test). Download Figure 4-1, TIF file.

### Morphologic features of axon-coupled spines

**Figure 5.**
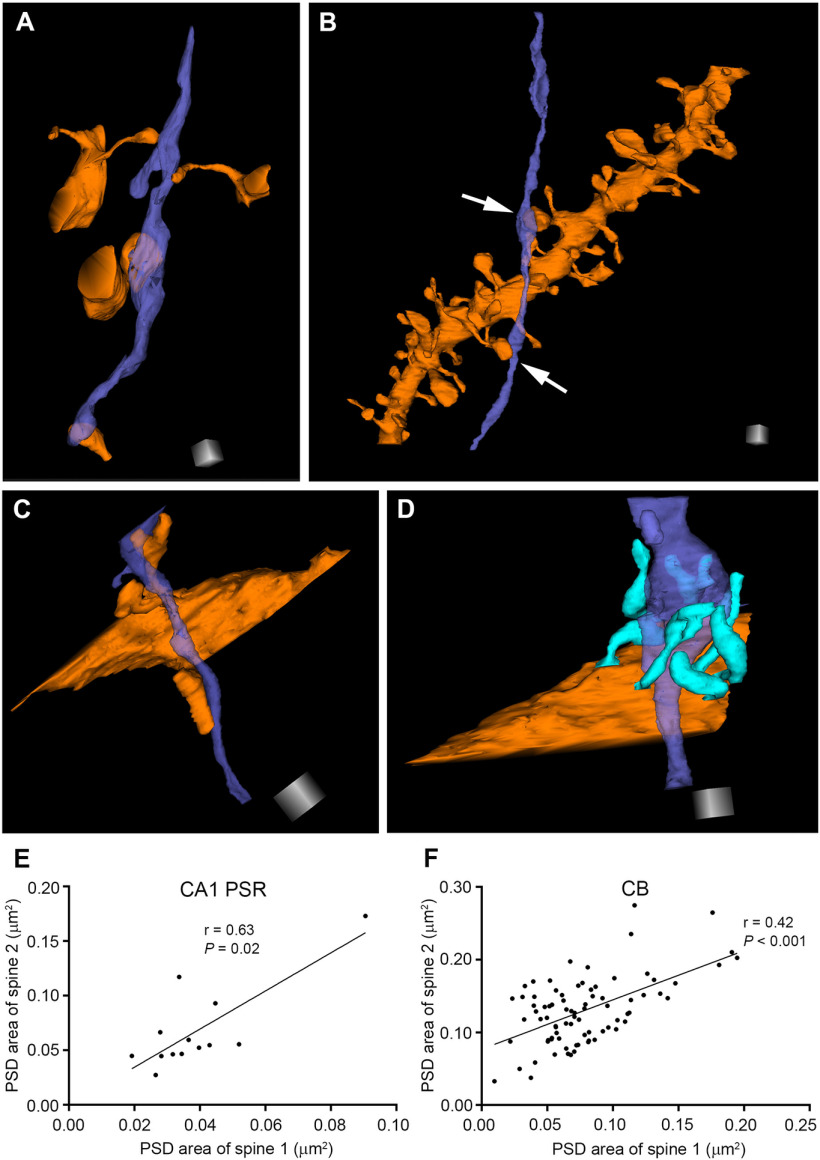
Intrapair synapse sizes are similar in axon-coupled spine pairs. ***A***, 3D reconstruction of a cortical axon (medium slate blue) that makes synaptic contacts with spines from multiple dendrites. ***B***, An example of an axon in the CA1 PSR that makes synaptic contacts with two spines (shown by white arrows) from the same dendrite. ***C***, An example of a parallel fiber in CB making synaptic contacts with three different spines from the same dendrite. ***D***, An example of a climbing fiber in CB making synaptic contacts with seven different spines (turquoise protrusions from the dendrite) from the same dendrite. PSD areas of axon-coupled spine pairs are positively correlated in the CA1 PSR (***E***) and CB (***F***). Scale cubes: 0.5 μm for each side.

We next analyzed whether the frequency of axon-coupled spines (spines that reside in the same dendritic segment and receive synaptic inputs from the same axon) correlates with the dendritic diameter. Axon-coupled spines are of special interest as they experience the same presynaptic inputs and postsynaptic membrane depolarizations, leading to a highly synchronized activation of signaling pathways related to spine structural changes. An increasing number of studies have shown that spatially close inputs onto spines summate non-linearly and increase the dendritic membrane potential beyond the threshold required for the generation of local dendritic spikes (Gasparini et al., 2004; Govindarajan et al., 2006; Larkum and Nevian, 2008). Thus, a higher frequency of axon-coupled spines can also result in higher synaptic strength in a particular dendritic segment. We followed the presynaptic terminals of each spine along the entire length of the dendritic tree contained within the imaging volume (Fig. 5*A–D*). Subsequently, we counted the frequency that the identified axons contact multiple spines located in the same dendrite. The frequency of axon-coupled spines was 4.7% in CA1 PSR, 25.7% in CA1 SLM, 5.4% in the cortex, 8.9% in the striatum, and 26.1% in CB. Interestingly, the canonical view that there exists a one-to-one connection between a parallel fiber to Purkinje cell is violated in >25% of the spines (Table 4). To examine whether the dendritic diameter can predict the relative frequency of axon-coupled spines, we plotted the frequency of axon-coupled spines against dendritic diameter. The plot revealed no significant correlation between these two parameters in CA1 PSR, CA1 SLM, striatum and CB (CA1 PSR: *r* = 0.46, *p *=* *0.07; CA1 SLM: *r* = 0.28, *p *=* *0.38; striatum: *r* = −0.07, *p *=* *0.78; CB: *r* = 0.06, *p *=* *0.85; Spearman’s rank order test). This analysis was not applied to the cortex, as only four dendrites contained axon-coupled spines. Our data demonstrate that the dendritic diameter cannot predict the frequency of the axon-coupled spines. Studies in CA1 PSR (Bartol et al., 2015), CA1 SLM (Bloss et al., 2018), and cortex (Kasthuri et al., 2015) have reported that the axon-coupled spines are of similar head volume. We further extended this finding and show that the axon-coupled spines have similar PSD areas in CA1 PSR (Fig. 5*E*) and CB (Fig. 5*F*). The variance of PSD size was smaller in axon-coupled spines compared with the rest of the spines both in CA1 PSR (variance in axon-coupled spines = 0.0008, rest of the spines = 0.0011) and CB (variance in axon-coupled spines = 0.00209, rest of the spines = 0.00257).

**Table 4:** Frequency of axon-coupled spines in different brain regions

Brain region	Spines observed (*n*)	Axon-coupled spines (*n*)	Number of spines in a dendrite making synaptic contacts with the same axon (range)
CA1 PSR	552	26 (4.7%)	2
CA1 SLM	70	18 (25.7%)	2
Cortex	221	12 (5.4%)	2
Striatum	403	36 (8.9%)	2
CB	824	215 (26.1%)	2–4

Note that all the 824 spines studied in CB make synaptic contacts with the parallel fiber terminals.

### Intracellular organelles in dendrites and spines

**Figure 6.**
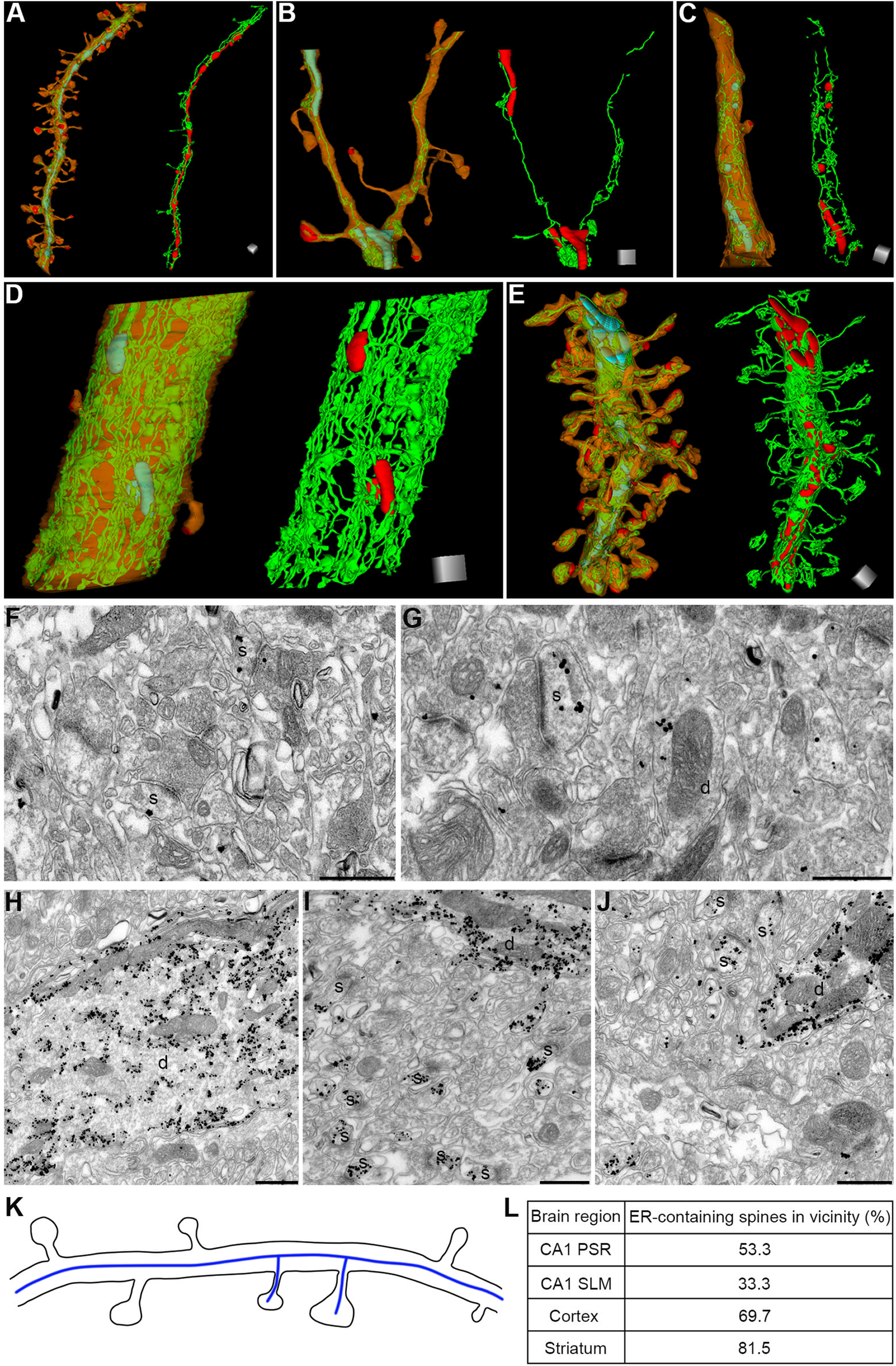
Distribution of ER in dendrites and spines. 3D reconstruction showing ER (green) and mitochondria (turquoise) in an oblique dendrite in the CA1 PSR (***A***), cortical dendrite (***B***), striatal dendrite (***C***), large-caliber dendrite in the CA1 PSR (***D***), and Purkinje cell dendrite in the CB (***E***). ER (green) is a continuous structure along the dendrite (orange) and extends into spines in all the brain regions. Dendrites, spines, PSDs (red), ER (green), and mitochondria (turquoise) are shown on the left-hand side, and only ER (green) and mitochondria (red) for the same dendrite are shown on the right-hand side in each panel in ***A–E***. TEM images showing immunoreactivity for IP_3_R1 in the dendrites (d) and spines (s) of the cortex (***F***), striatum (***G***), and CB (***H–J***). The schematic diagram in ***K*** shows that the ER (blue line) is continuous along the dendrite and extends into spines. Panel ***L*** shows that in >50% of the cases, ER-containing spines are located next to one another in the CA1 PSR, cortex, and striatum. Scale cubes: 0.5 μm (***A–E***, for each side). Scale bars: 500 nm (***F–J***).

Lastly, we looked into the distribution of ER in the spines. In all the brain regions that we have studied, we found that ER is a continuous structure along the dendrites and extends into the spines (Fig. 6*A–E*). The frequency of spines positive for ER varies depending on the brain region (Table 5). ER-containing spines had significantly larger head volume and wider neck diameter in CA1 PSR, CA1 SLM, cortex, and striatum (Table 5). However, the neck length did not differ significantly between those spines that contained ER and those that did not.

**Table 5:** Distribution of ER in spines

Brain region	Spines observed (*n*)	ER-containing spines (%)	Head volume (mean ± SD)		Neck length (mean ± SD)		Neck diameter (mean ± SD)	
			ER+	ER–	ER+	ER–	ER+	ER–
CA1 PSR	552	13.6	0.11 ± 0.073	0.04 ± 0.030***	0.46 ± 0.229	0.46 ± 0.249	0.25 ± 0.101	0.19 ± 0.082***
CA1 SLM	70	15.7	0.18 ± 0.077	0.11 ± 0.084*	0.55 ± 0.233	0.48 ± 0.300	0.34 ± 0.090	0.23 ± 0.127**
Cortex	221	40.3	0.15 ± 0.131	0.04 ± 0.023***	1.03 ± 0.566	1.13 ± 0.557	0.30 ± 0.143	0.19 ± 0.075***
Striatum	403	55.1	0.10 ± 0.137	0.02 ± 0.017***	1.15 ± 0.584	1.09 ± 0.518	0.29 ± 0.105	0.21 ± 0.085***
CB	824	100	—	—	—	—	—	—

Units: head volume, μm^3^; neck length, μm; neck diameter, μm.

Asterisks denote statistical significance in the dimensions of head volume and neck diameter between ER-containing (ER+) and ER-lacking spines (ER–) in each brain region.

To further corroborate the finding from FIB/SEM images on the frequency of ER-containing spines in the cortex, striatum, and CB, we performed pre-embedding immunogold labeling with an antibody against IP_3_R1 (Fig. 6*F–J*). We observed that all the spines showed immunoreactivity for IP_3_R1 in CB. However, in the case of cortex and striatum, we found that the percentages of IP_3_R1 positive spines were somewhat lower than the percentages of spines estimated to contain ER based on FIB/SEM images (frequency of IP_3_R1 positive spines in cortex = 30.2%, striatum = 35.2%; frequency of ER-containing spines in cortex = 40.3%, striatum = 55.1%). This is not unexpected because IP_3_R1 content is much lower in the dendrites and spines of cortex and striatum than in the dendrites and spines of CB (Fig. 6*F–J*). As the labeling efficiency of the pre-embedding immunogold labeling method is unlikely to be 100% because of the low number of IP_3_R1 in individual spines, the frequency of spines containing IP_3_R1 is likely to be somewhat under-represented in the case of cortex and striatum.

Interestingly, a significant fraction of ER-containing spines is often located in the vicinity (53.3% of spines in CA1 PSR, 33.3% of the spines in CA1 SLM, 69.7% of spines in the cortex, 81.5% of spines in the striatum; Fig. 6*K*,*L*), suggesting that the ER-containing spines form a hotspot along the dendritic segment.

## Discussion

Morphology serves as the basis for physiological functioning. Here, using FIB/SEM to perform volume imaging from several brain regions, we provide a thorough overview of dendritic spine morphology. The use of FIB/SEM overcomes inherent problems associated with serial section TEM (ssTEM) such as variations in section thickness and image distortion during sectioning and image acquisition. Manual segmentation of structures provides a more accurate reflection of the spine dimension compared with the automated machine-learning based segmentation. Cross comparison of different brain regions was performed from the same brain, thus ascertaining uniformity in terms of age, sample preparation, and methods of analysis. Our reconstruction data of a large number of dendrites and their dendritic spines show that the dendritic spines, despite their great structural diversity, are organized along a dendrite in a regulated manner. The breadth of data provided by our study can serve as a reference for the functional interpretation of various experimental data acquired by synaptic physiologists.

### Regional uniqueness in spine morphology

Even after an extensive analysis of hundreds of spines, to accurately identify the brain region where a given spine was sampled might still be a daunting task. High variability and significant overlap in spine dimensions across brain regions preclude identification of a structural fingerprint unique to each area. However, with a certain degree of confidence, spines with large head volume and short neck length can be associated with CA1 SLM; large head volume and medium neck length with CB; medium head volume and long neck length with cortex; medium head volume, long and swollen wide necks in the midway with striatum; and small head volume and short neck length with CA1 PSR. Moreover, incorporation of other structural parameters in the dendrites, such as spine density, frequency of branching, spine perforation, etc. can aid in identifying the brain region where a spine is sampled.

Short spine necks facilitate rapid transfer of electrical (Araya et al., 2006b, 2014; Fig. 4) and chemical (Svoboda et al., 1996) signals from the spine head toward the dendritic shaft. Spine necks in CA1 PSR and CB are relatively shorter than the other brain regions that we have studied. Besides, the dendrites in these two areas possess high spine density (Fig. 2*F*; Table 2). Short spine necks and small inter-spine distance create a favorable situation for signal cross talk among spines. Indeed, by two-photon glutamate uncaging and time-lapse imaging, heterosynaptic interaction between CA1 PSR spines has been directly demonstrated (Oh et al., 2015). It would be interesting to examine whether heterosynaptic interactions could be induced more efficiently and reliably in CA1 PSR and CB than in CA1 SLM, cortex, and striatum.

Unlike in the CB and CA1, the spine heads of the cortex and striatum receive multiple presynaptic inputs with distinct neurotransmitter contents from various brain nuclei. The long necks in the cortex and striatum hinder the diffusion of signals from the spine heads toward the dendritic shafts and retain the signal within a spine head for a considerable amount of time. As a result, this increases the time-window for intraspine interaction of afferent signals that use different neurotransmitters and originate from multiple brain areas. The wider time-window of signal interaction is particularly useful in the striatum, where the communication of dopaminergic and glutamatergic inputs is necessary to drive reward learning (Yagishita et al., 2014).

### Common synaptic organizational principle

We revealed that the PSD area per unit length of dendrite increases as a function of dendritic diameter. One may argue that this is not unexpected as bigger dendrites, compared with the smaller ones, contain larger membrane surface area and resources necessary to generate more PSD structures. However, the fact that this trend is not observed in the striatum and CA1 SLM suggests that the availability of higher membrane surface area in dendrites alone cannot account for the gradient in PSD density. We believe that this type of organizational principle is unique for dendrites in CA1, CB, and cortex, whose somata are organized in discrete layers.

What function does it serve to have intradendritic scaling of PSD density? One possibility is that the intradendritic scaling plays a role to normalize membrane depolarization by creating a counterbalance between the magnitude of synaptic inputs and the input impedance of the dendritic shaft. Thin dendrites require relatively few inputs to generate enough depolarization. In the case of thick dendrites, a large number of stronger inputs are necessary to create dendritic spikes. Therefore, it is physiologically relevant to have a high number of big synapses in the large-diameter dendrites. Perhaps intracellular organelles that are abundant in thick-diameter dendrites facilitate an increase in the PSD area. A large fraction of spines contains ER in multiple brain regions, and ER-containing spines have larger spine heads (Table 5). On the other hand, spine neck length is not positively regulated by spine ER, suggesting independent regulatory pathways of spine head enlargement and spine neck growth, both of which contribute to the local regulation of synaptic strength.

We also identified a positive correlation between the spine neck length and the dendrite diameter. This observation rather supports the idea that higher PSD density and longer spine neck length compensate with each other in thick dendrites and result in a less noticeable difference in total synaptic strength between the thin and thick dendrites. Synaptic constancy across dendrites suggests that although the synaptic strength of individual spines or synaptic connections dynamically changes as a result of synaptic plasticity, the total synaptic strength in the dendrite remains constant. This synaptic constancy hints at the existence of a homeostatic mechanism at the level of individual dendritic branches that compensate for changes in the synaptic weights of individual spines. Using ssTEM images, Bourne and Harris (2011) revealed that on long-term potentiation, the stimulated spines grew in volume at the expense of unstimulated spines such that the PSD area per unit dendritic length remained conserved between control and stimulated conditions. What could be the functional relevance of maintaining synaptic constancy among dendritic branches? Our computational study indicates that synaptic constancy among CA1 dendrites enables them to integrate a moderate level of synaptic noise to the authentic synaptic signal and enhance the overall signal transmission from the strongest synapse toward the soma. Constancy in synaptic strength may also be beneficial for the coordinated firing of CA1 place cells during θ oscillations (Buzsáki, 2002) as these cells use phase-dependent firing activity relative to background oscillatory inputs for information processing.

Our electrophysiology data showed that the uncaging EPSP amplitudes at the soma are independent of the spine head widths, but are rather inversely correlated with the spine neck lengths. As a result, it can be expected that EPSPs produced by spines located in the large-diameter dendrites would be attenuated more severely because of their longer necks. The differential filtering of synaptic potentials may serve a purpose to normalize the EPSP amplitude at the soma regardless of the site of its origin in the dendrite.

The integrative property of dendrites is heavily influenced by their distance from the cell body. Thus, the precise distance of each spine and dendrite is highly informative in understanding their relative contribution in somatic depolarization. In this study, we did not attempt to measure the precise distance of each dendrite and spine from their respective cell bodies, which require time-consuming correlative light and EM (CLEM) and retrospective EM reconstruction. In general, dendritic diameter serves as a reliable indicator of the relative distance of the individual dendrites from their parent soma (Grillo et al., 2018). However, in our data from CA1 PSR, the dendritic diameter may not necessarily reflect the distances of the dendrites from their soma. Since the imaging volume in CA1 PSR was located at a distance of ∼100 μm from the edge of the CA1 pyramidal cell layer, the straight-line distances of dendrites do not vary considerably from their corresponding cell bodies. Thus, rather than the distance from the soma, the dendritic diameter may indicate the relative distance from the dendritic branch points on the primary apical dendrite. As shown previously (Menon et al., 2013; Benavides-Piccione et al., 2020), we surmise that the thick dendrites in our datasets represent dendritic segments near the branch points, and thin dendrites represent dendritic segments away from the branch points.

The spine distribution pattern in hippocampal CA1 dendrites is likely conserved across species and different developmental stages. Somewhat similar to our findings, Katz et al. (2009) have previously shown that in the apical oblique dendritic branches of six-month-old rats, both the spine density and the average synapse size decrease toward the distal ends. Interestingly, in the basal dendrites in the CA1 stratum oriens of six-month-old rats, the spine volume was significantly smaller, and the spine density was significantly lower in the small-diameter dendrites than that in the large-diameter dendrites (Menon et al., 2013). In another study in P21 mice (Walker et al., 2017), it was shown that the active zone area of the presynaptic bouton, the volume, and the PSD area of dendritic spines scales positively with the dendritic diameter. Similarly, it was shown that both in the P22 and P100 mice, the size of the presynaptic active zone increases with the dendritic diameter and decreases with the distance from the cell body (Grillo et al., 2018). These studies, taken together, suggest that the organizational principle of dendritic spines in the CA1 pyramidal cells is similar both in the apical oblique and the basal dendrites and is maintained throughout developmental stages both in the mice and rats.

Previous studies in CA1 PSR (Bartol et al., 2015), CA1 SLM (Bloss et al., 2018), and cortex (Kasthuri et al., 2015) have shown that the axon-coupled spines have similar head volumes and neck lengths. We show that this observation holds in the CB as well, suggesting that this may be one of the conserved principles underlying axon-spine connectivity. The fact that axon-coupled spines have similar PSD areas hints that these spines have the same history of plasticity. We also revealed that ER-containing spines are located in the vicinity to form a hotspot. Moreover, in comparison to ER-lacking spines, ER-containing spines have larger heads and wider necks. Neuronal activity or plasticity-related stimulus may serve as the address marker for dictating which specific spine should harbor ER. Indeed, Imaging experiments *in vitro* and *in vivo* have shown that experience results in the activation of nearby spines located in the same branchlet (Takahashi et al., 2012; Lee et al., 2016; Frank et al., 2018) and the potentiated spines have larger heads and wider necks than the un-potentiated spines (Fifková and Anderson, 1981). Perhaps, ER is selectively recruited to such potentiated spines to sustain an increased demand for calcium ions following synaptic potentiation.

In conclusion, our study provides an accurate morphologic description and physiological role of various parameters that shape synaptic transmission and the principle underlying the placement of dendritic spines in a dendritic branch. Ultimately, with the development of a high throughput image acquisition and image analysis method, it is necessary to extrapolate these findings to all the spiny neurons in the brain. Although a great deal of labor is expended for a study of this nature, the wealth of information that it provides is rewarding.

## Synthesis

Reviewing Editor: Juan Burrone, King’s College London Decisions are customarily a result of the Reviewing Editor and the peer reviewers coming together and discussing their recommendations until a consensus is reached. When revisions are invited, a fact-based synthesis statement explaining their decision and outlining what is needed to prepare a revision will be listed below. The following reviewer(s) agreed to reveal their identity: Zoltan Nusser, Masahiko Watanabe.

The authors use multiple techniques to assess the properties of spines along the dendrites of neurons in multiple brain areas. They provide high-quality FIB/SEM data sets that allow them to characterise the morphology of spines and relate them to the properties of the dendrites they belong to. They then use electrophysiology and glutamate uncaging to measure spine function and relate these measures to morphology. Finally, they use mathematical modelling to place their findings in the context of dendritic integration. Although their findings are interesting, there are a number of issues raised by the reviewers that need to be addressed at this stage. Below are the comments raised by the reviewers in full.

Reviewer 1

The present MS describes experiments aiming at revealing principles in the size and geometry of dendritic spines in many different brain regions. The authors performed high quality FIB/SEM experiments; two-photon glutamate uncaging and patch-clamp recordings to reveal a number of important principles in the geometry of spines. Although, the MS contains important, high-quality data, there are a number of shortcomings of the design. The most important is that the authors are unable to tell how far a given dendrite is from the soma. They cannot analyze within cell and among cell variability for a given dendritic diameter. Sparse viral expression of a marker protein (e.g. eGFP) and post-hoc reconstruction of individual cells would be essential for unequivocal identification of dendrites at certain distance from the soma.

In the result section, the authors argued that the spine neck length should be a major determinant of the voltage-attenuation to the soma. Why is that more important than the neck diameter? In order to verify this, they performed two-photon glutamate uncaging and somatic recording. These type of experiments have been performed many times and provided conflicting data. One of the reasons is that the precise geometry, especially that of the spine neck cannot be determined with 2P LM. This problem is also present in this study.

The authors report that the spine neck length influences the uEPSP amplitude at the soma, but the head diameter is not. This comes from grouping spines with similar head size and different neck length and similar length with different head sizes. The authors report a neck length of ˜0.1 um. How could the authors measure a distance well below the diffraction limit? Similar, how do the authors know that two spines that have seemingly the same size are indeed similar rather than just the consequence of the diffraction limit?

The most likely explanation from the lack of correlation between the postsynaptic response size and the spine head diameter is that the authors activate a very large pool of extrasynaptic receptors on the spines/neck/dendritic shafts due to the large laser power and large uncaging spot. Indeed, an uncaging spot closer to the dendrite would activate more extrasynaptic dendritic AMPARs and would result in a larger EPSP compared to the spot that is ˜1 um away of the dendrite. This would be (mis)interpreted as the different spine neck lengths filtered the response. This is consistent with the extremely long uncaging pulse!

The authors write: ‘Since this second population (dendritic diameter > 1.4 um) is highly likely to be contaminated by the dendrites of the main apical trunk, which are known to have very few to no spines,’. Why is it a problem? And if such dendrites are present in their sample, their very low spine density should make them a separate population, allowing the authors to subselect them.

They also state: ‘ Nevertheless, our data demonstrate that the dendritic diameter can accurately predict the PSD content in CA1 PSR,..’. This is clearly not the case, given the large variance. A significant correlation does not mean that one parameter ‘accurately’ predict the other.

They state: ‘ These facts suggest that, despite the positive relationship of the spine head volume and the neck length with the dendritic diameter, synaptic strength is maintained relatively constant irrespective of the dendritic diameter in CA1 PSR.’ That is clearly an oversimplification. That is only true if both parameter had the same correlation and their functional consequence would be identical (e.g. both linear with the same slope!).

The authors examined spines that receive input from the same axon. They should have calculated the variance in the PSD size among such spines and compare that to the whole population.

Minor points:

The authors mention that CA3 PSR is also included in the study. The reviewer cannot see that data.

In Fig 2E where the authors ‘clustered’ the different spiny cells they used a dendrite as a unit. Why?

In the result section, where the authors describe the results of the spine head volume, they only mention the statistical tests, but not the actual values (unlike for the spine neck length just below). The reviewer feels that it would be beneficial to provide the mean, SD values in the text.

In all tables, please provide SD rather than SEM.

‘frequency of IP3R1 positive spines in cortex = 30%, striatum = 35%; compare this with the values in Table 6-1).’ It is cumbersome to compare values is one value is given in the text and the other is somewhere in a table. For such comparisons, please provide the values in the text.

Reviewer 2

Using volume EM, two-photon imaging, glutamate uncaging, electrophysiology, and computer simulation, this study (eN-NWR-0248-20) investigated the fundamental anatomical organization of dendritic spines in several higher brain regions of mice, including the hippocampus, cortex, striatum, and cerebellum. Volume EM analyses by FIB/SEM elucidated that, despite different spine geometry and dimensions across neuronal cell types, the PSD area per unit length of dendrites and the spine neck length are positively correlated with the diameter of dendrites in several brain regions having layered cortical organization. This finding suggests that the ratio of the PSD area and spine neck length is relatively uniform across dendrites with different diameters. To this point, computer simulation suggests that this uniformness is effective and advantageous for synaptic input transfer from dendrites to the soma. Glutamate uncaging and electrophysiological assessment revealed that the spine neck length is more influential than spine head size in regulating the EPSP magnitude at the soma.

This study is constructed in well-organized and appropriate manners. I raise minor comments only, for further improvement.

1. Dendrites with <1 um in diameter were sampled from CA1 PSR to prevent contamination of the main apical trunk having very few or no spines (Extended figure 2-3). Likewise, Purkinje cell dendrites appear to be sampled from distal spiny dendrites, as the mean diameter is 1 um (Table 2-2). Information of the sampling criteria should be described in the Method section or Results.

2. In Table 1-1, spines in branches are presented as 2, 2-3, 2-4, or 2-5. Please explain what these scores stand for.

3. In Table 2-1, the last sentence of the legend “One large diameter dendrite did not contain any dendritic spine” can not be understood. Was it noted to mean that this dendrite was eliminated from the analysis?

4. To measure the spine volume, fluorescent proteins should have been introduced to organotypic slice cultures. This procedure is not described in the Method section.

5. In Fig. 2, box plots display the median value and certain percentiles. What percentiles are adopted?

6. Extended figure 2-1 is composed of A through J panels to a left-to-right direction (AB, CD, EF, GH, and IJ). This figure will become easier to understand if A-E and F-J are placed longitudinally, by which graphs of the PSD area and neck length in the same regions line up next to each other.

7. Abbreviation of CB (cerebellum) and SLM (stratum lacunosum-moleculare) are used without spelling out. Also, cerebellum is used at some place after use of abbreviation of CB.

8. Almost all the spiny protrusions displayed synaptic contacts with their presynaptic partner (Table 1-1). This sentence is at line 316-317. However, this table does not indicate the information.

9. In line 434. Fig.2D should be cited such that “neck length in individual spines is similar between CA1 PSR and CB (Fig.2D)”.

10. In line 629-631, Table 5-1 does not include the information showing that one to one connection between parallel fiber to Purkinje cell synapse is violated in 25% of the spines.
